# Single cell-derived multicellular meristem: insights into male-to-hermaphrodite conversion and *de novo* meristem formation in *Ceratopteris*

**DOI:** 10.1242/dev.204411

**Published:** 2025-02-13

**Authors:** Xi Yang, An Yan, Xing Liu, Alexandria Volkening, Yun Zhou

**Affiliations:** ^1^ Department of Botany and Plant Pathology, Purdue University, West Lafayette, IN 47907, USA; ^2^ Purdue Center for Plant Biology, Purdue University, West Lafayette, IN 47907, USA; ^3^ Division of Biology and Biological Engineering, California Institute of Technology, Pasadena, CA 91125, USA; ^4^ Howard Hughes Medical Institute, California Institute of Technology, Pasadena, CA 91125, USA; ^5^ Department of Biochemistry, Purdue University, West Lafayette, IN 47907, USA; ^6^ Department of Mathematics, Purdue University, West Lafayette, IN 47907, USA

**Keywords:** Cell division, Hermaphroditic gametophyte, Male gametophyte, Multicellular meristem, Seed-free plants, Ferns, Population model, *De novo* meristem formation, *Ceratopteris*

## Abstract

Land plants alternate between asexual sporophytes and sexual gametophytes. Unlike seed plants, ferns develop free-living gametophytes. Gametophytes of the model fern *Ceratopteris* exhibit two sex types: hermaphrodites with pluripotent meristems and males lacking meristems. In the absence of the pheromone antheridiogen, males convert to hermaphrodites by forming *de novo* meristems, although the mechanisms remain unclear. Using long-term time-lapse imaging and computational analyses, we captured male-to-hermaphrodite conversion at single-cell resolution and reconstructed the lineage and division atlas of newly formed meristems. Lineage tracing revealed that the *de novo*-formed meristem originates from a single non-antheridium cell: the meristem progenitor cell (MPC). During conversion, the MPC lineage showed increased mitotic activity, with marginal cells proliferating faster than inner cells. A mathematical model suggested that stochastic variation in cell division, combined with strong inhibitory signals from dividing marginal cells, is sufficient to explain gametophyte dynamics. Experimental disruption of division timing agreed with the model, showing that precise cell cycle progression is essential for MPC establishment and sex-type conversion. These findings reveal cellular mechanisms governing sex conversion and *de novo* meristem formation in land plants.

## Teaser

**Summary:** Long-term time-lapse imaging and computational analyses reveal that cell division dynamics drive the *de novo* formation of single cell-derived multicellular meristems during sex-type conversion in *Ceratopteris* gametophytes.

## INTRODUCTION

Land plants undergo alternating generations of asexual sporophytes and sexual gametophytes (Bowman et al., 2016; Jill Harrison, 2017). In seed plants, sporophytes develop apical and lateral meristems to drive organ formation and shape plant architecture, while their gametophytes lack meristems and rely on sporophytes for growth (Meyerowitz, 1997; Steeves and Sussex, 1989; Yadegari and Drews, 2004; McCormick, 2004; Li and Ma, 2002). Unlike seed plants, ferns exhibit independent growth in both sporophytes and gametophytes, and fern gametophytes are free-living, maintaining pluripotent meristems to sustain prothallus expansion and sexual organ development (Banks, 1999; Nayar and Kaur, 1971; Plackett et al., 2015; Wu et al., 2023; Imaichi, 2013). Gametophytes of diverse fern species develop distinct types of meristems, including apical cell (AC)-based meristems and multicellular meristems (Wu et al., 2023; Imaichi, 2013). The AC-based meristem comprises a wedge-shaped AC and its progeny, typically located at the apical region of the prothallus, driving cell proliferation (Wu et al., 2023; Imaichi, 2013; Takahashi et al., 2009; Wu et al., 2022a,b). Multicellular meristems, by contrast, exhibit different morphology and organization, consisting of an array of actively dividing, rectangular cells with small dimensions (Imaichi, 2013; Takahashi et al., 2009; Wu et al., 2022a; Plackett et al., 2014). Among them, the multicellular apical meristem is located at the prothallus apex (Takahashi et al., 2009; Wu et al., 2022a), while the multicellular marginal meristem (Wu et al., 2023), also referred to as the marginal meristem (Geng et al., 2022; Bartz and Gola, 2018), multicellular meristem (Imaichi, 2013; Takahashi et al., 2012) or lateral meristem (Banks, 1999; Banks et al., 1993) in previous studies, originates from one lateral side of the prothallus.

The homosporous fern *Ceratopteris richardii* has been widely used as a model organism to address various biological questions in ferns (Banks, 1999; Plackett et al., 2015, 2018; Hou and Hill, 2002; McAdam et al., 2016; Bui et al., 2017; Youngstrom et al., 2019; Conway and Di Stilio, 2020; Geng et al., 2021, 2024a; Marchant et al., 2019, 2022; Kinosian and Wolf, 2022; Hickok et al., 1995, 1987). *Ceratopteris* alternates between haploid gametophytes and diploid sporophytes. Its gametophytes display two distinct sex types: hermaphroditic and male (Fig. S1). These sex types are determined post-spore germination by the pheromone antheridiogen, which is produced by hermaphroditic gametophytes (hermaphrodites) and released into the environment to induce male programming in neighboring gametophytes (Hickok et al., 1995, 1987) (Fig. S1). Specifically, in the absence of antheridiogen, *Ceratopteris* spores develop as hermaphrodites, each containing a multicellular meristem, several egg-bearing archegonia (female organs), and a few sperm-producing antheridia (male organs) (Banks, 1999; Banks et al., 1993; Hickok et al., 1987) (Fig. S1). The multicellular meristem in hermaphrodites remains undifferentiated and sustains cell division, resulting in an expanded, heart-shaped prothallus with a notch at the center (Banks, 1999; Banks et al., 1993). On the contrary, spores germinated in the presence of antheridiogen develop as male gametophytes, which are significantly smaller than hermaphrodites, lack a meristem identity, and quickly undergo a cellular differentiation program to produce a number of sperm-producing antheridia (Banks, 1999; Banks et al., 1993; Hickok et al., 1987) (Fig. S1). Previous studies have characterized and defined various developmental stages of *Ceratopteris* hermaphrodites and males (Bartz and Gola, 2018; Banks et al., 1993; Conway and Di Stilio, 2020). Because the meristem and prothallus of a hermaphrodite consist of a single layer of cells, which is well-suited for non-invasive live imaging, a recent study using time-lapse confocal imaging revealed the cell division dynamics driving meristem development in *Ceratopteris* hermaphrodites (Geng et al., 2022).

Gametophyte development in *Ceratopteris* exhibits significant plasticity, with sex types capable of switching in response to environmental signals and stimuli (Banks, 1999; Hickok et al., 1987). The maintenance of male developmental programming depends on antheridiogen. In the presence of antheridiogen, a male retains its male characteristics, promoting nearly all of the cells in its body to differentiate into antheridia, which eventually mature, rupture, and release motile sperm for fertilization. In contrast, in the absence of antheridiogen, a previously determined male undergoes a conversion into a hermaphrodite, accompanied by the *de novo* formation of a meristem and subsequent development of egg-producing archegonia adjacent to the newly formed meristem (Banks, 1999; Hickok et al., 1995). This naturally occurring process, triggered by the lack of antheridiogen, appears to play an essential role in maintaining a balance between males and hermaphrodites within a gametophyte population, thereby ensuring efficient and successful sexual reproduction (Hickok et al., 1995). Despite this understanding, the mechanisms driving this fascinating switch between two distinct sex types, as well as the cellular processes involved in the *de novo* formation of meristems from ameristic males, remain largely unknown.

In this study, we generated transgenic *Ceratopteris* lines expressing a new ubiquitous fluorescent reporter in both hermaphrodites and males. We then performed long-term, non-invasive time-lapse confocal imaging to examine the formation of *de novo* meristems during the male-to-hermaphrodite conversion. Using drug treatments, computational image segmentation, lineage tracing, and quantitative division analyses, we mapped the lineage dynamics and division activity during both normal and perturbed processes of the sex-type conversion. Our results demonstrate that a single non-antheridium cell in the male gametophyte acts as the progenitor of the entire *de novo*-formed meristem. Based on a simple mathematical model of cell population growth, we propose that this progenitor cell may arise through the combination of two dynamics: stochastic variations in cell division and strong inhibitory signals from actively dividing cells in the marginal layer. Taken together, these findings uncover cellular mechanisms driving *de novo* meristem formation in ferns, broaden our understanding in the regulation of sex conversion, and suggest new directions for future interdisciplinary research.

## RESULTS

### Emergence of multicellular meristems from ameristic males

**Fig. 1.**
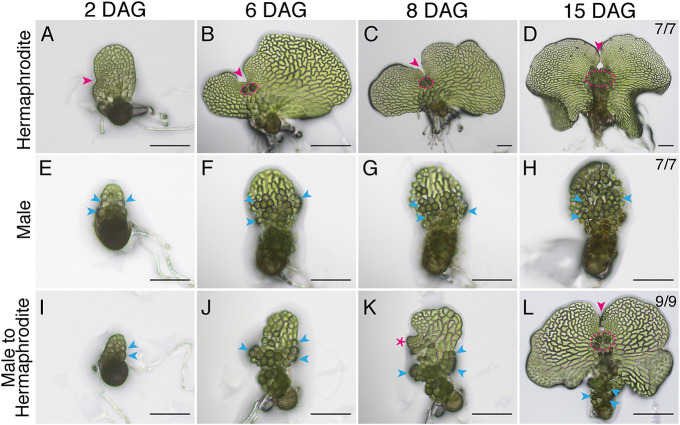
**Dynamic processes of hermaphrodite and male development, and *de novo* meristem formation during the male-to-hermaphrodite conversion in *Ceratopteris*.** (A-H) Light micrographs showing the developmental progression of a representative hermaphrodite (A-D) and a representative male (E-H) grown in the presence of antheridiogen. Images were taken at the indicated days after germination (DAG). This experiment included seven hermaphrodites and seven males, all showing comparable results within their respective sex types. (I-L) Light micrographs of a representative male transferred to an antheridiogen-free medium at 2 DAG, undergoing developmental conversion to a hermaphrodite over time. Images were taken at the time of transfer (I) and at the indicated DAG (J-L). Nine males were included in this experiment, all of which successfully underwent the male-to-hermaphrodite conversion by 15 DAG. Magenta arrowheads indicate the developing meristem in the hermaphrodite (A-D) and the *de novo* formation of a new meristem during the male-to-hermaphrodite conversion (L). (K) The magenta asterisk shows the site of active proliferation during the conversion. (B-D,L) Magenta dashed outlines highlight archegonia initiated adjacent to the meristem of the hermaphrodite (B-D) or the newly formed meristem (L). (E-L) Blue arrowheads highlight a few antheridia. Scale bars: 100 μm.

We first characterized the developmental changes during the male-to-hermaphrodite conversion and compared them to the typical developmental programs of meristic hermaphrodites and ameristic males (Fig. 1A-L, Fig. S1). Consistent with previous observations (Banks et al., 1993), males and hermaphrodites were morphologically distinguishable by 2 days after germination (2 DAG) (Fig. 1A,E,I). At this stage, hermaphrodites had initiated a multicellular meristem (magenta arrowhead, Fig. 1A), while males lacked a meristem and began forming differentiated, sperm-producing antheridia (blue arrows, Fig. 1E,I). Once established (Fig. 1A), the meristem in the hermaphrodite continually proliferated, resulting in a greatly expanded, heart-shaped prothallus with a characteristic meristem notch (Fig. 1B-D, magenta arrowheads) and several egg-bearing archegonia (Fig. 1B-D, magenta dashed circles). Unlike hermaphrodites, in response to antheridiogen, the male maintained its sex type and continued the differentiation of sperm-producing antheridia (Fig. 1F-H, blue arrowheads). In contrast, when a male gametophyte (2 DAG) was transferred to an antheridiogen-free growth medium, it gradually lost its male identity (Fig. 1I-L). Several days after the transfer, the male gametophyte developed a new active proliferation site (Fig. 1K, magenta asterisk), which eventually formed a new hermaphrodite converted from the original male body (Fig. 1L). Although the converted hermaphrodite (Fig. 1L) was smaller than the continuously proliferating hermaphrodite (Fig. 1D) after meristem formation (Fig. 1A), its morphology was distinct from the male at the same DAG (Fig. 1H) and highly comparable to the hermaphrodite (Fig. 1B-D), with a *de novo*-formed meristem (Fig. 1L, magenta arrowhead) and several adjacent archegonia (Fig. 1L, magenta dashed circle).

### Long-term time-lapse imaging reveals cell division dynamics during male-to-hermaphrodite conversion

We aimed to perform confocal live imaging to examine *de novo* meristem formation during the male-to-hermaphrodite conversion at single-cell resolution. To visualize individual cells, we generated a new transgenic *Ceratopteris* line expressing a ubiquitously expressed nuclear marker (*pCrUBQ10::H2B-GFP::3′CrUBQ10*). In this construct, Histone-2B fused with GFP (H2B-GFP) was placed under the control of the promoter and 3′ terminator of one *Ceratopteris Ubiquitin 10* (*CrUBQ10*) gene (Fig. S2A-F). After stably transforming this reporter construct into *Ceratopteris*, we obtained multiple independent transgenic lines showing comparable reporter expression in gametophytes (Figs S2 and S3). Laser scanning confocal imaging revealed strong and ubiquitous expression of the reporter in all cell types, including meristems, egg-producing archegonia, sperm-producing antheridia, and gametes in both males and hermaphrodites (Figs S2A-F and S3A-H). Furthermore, this reporter maintained high and consistent expression in the following generation of *Ceratopteris* gametophytes. These results confirmed that our transgenic lines are well-suited for monitoring cell division dynamics during the male-to-hermaphrodite conversion.

**Fig. 2.**
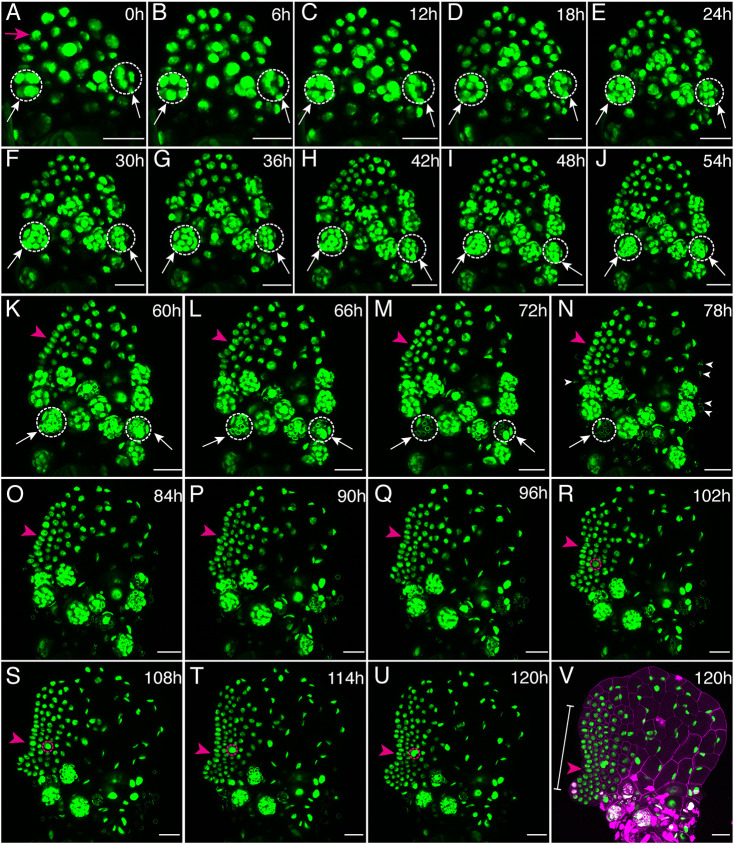
**Time-lapse confocal imaging of the male-to-hermaphrodite conversion in *Ceratopteris*.** (A-V) *Z*-projection views of one *Ceratopteris* gametophyte (Sample 1) expressing the *pCrUBQ10::H2B-GFP::3′CrUBQ10* transgenic reporter. (A-U) At 2 DAG, the male gametophyte was transferred from conditioned fern medium (CFM, with antheridiogen) to FM (without antheridiogen) and imaged by laser confocal microscopy immediately after transfer (0 h). The gametophyte was live-imaged every 6 h for up to 120 h, capturing its conversion into a hermaphrodite with a meristem notch and adjacent archegonia. Signals from the GFP channel are shown. (A-N) White dashed circles and white arrows highlight two representative antheridia from initiation to maturation (releasing sperm). (N) White arrowheads (78 h) indicate motile sperm released from antheridia. (A) The magenta arrow indicates the progenitor cell contributing to the formation of a new meristem; (K-V) magenta arrowheads indicate the formation of a meristem notch; (R-U) magenta circles highlight the initiation and development of egg-producing archegonia. (V) The same sample at 120 h as in U, showing merged signals from the GFP channel (green) and PI counterstain (magenta, showing the cell outline). Scale bars: 50 μm. At least three samples were live-imaged with the same settings and time intervals, showing comparable results. Two other independent replicates are included in Figs S4 and S5, respectively.

We then performed long-term, non-invasive time-lapse imaging of this reporter using laser scanning confocal microscopy to examine cell behavior during the dynamic male-to-hermaphrodite conversion process (Fig. 2). In brief, transgenic spores were surface-sterilized and inoculated on growth medium supplemented with antheridiogen, ensuring that germinated gametophytes developed into males. Two days after germination, males were selected and transferred to fresh growth medium without supplemented antheridiogen to induce the conversion to hermaphrodites. Imaging began immediately following the transfer, marking the first time point (0 hours, 0 h) (Fig. 2A). The samples were then cultured under the same growth conditions, with live imaging repeated every 6 h until new meristems were fully established (Fig. 2B-V).

The time-lapse confocal images (Fig. 2) revealed that, following the removal of antheridiogen from the growth media, *Ceratopteris* males underwent a gradual morphological transformation and activated the division program for non-antheridium cells. Specifically, during the first 54 h (Fig. 2A-J), several non-antheridium cells divided, resulting in a cluster of newly formed cells/nuclei primarily located at the apical part of the male. By 60 h, the initiation and development of a new meristem became apparent (Fig. 2K, highlighted by the magenta arrowhead), characterized by an array of recently divided cells in the marginal layer. From then on, the newly formed meristem continued to proliferate (Fig. 2K-V, magenta arrowheads), constantly increasing cell numbers and gradually forming the typical concave structure known as the meristem notch. Concurrent with the establishment of the *de novo* meristem, egg-producing archegonia (female organs) (Fig. 2R-V, highlighted with magenta dashed circles) began to form and develop adjacent to the meristem notch, closely resembling the typical developmental pattern seen in *Ceratopteris* hermaphrodites (Geng et al., 2022). Meanwhile, the original male body continued its pre-existing antheridium developmental program and eventually completed it (Fig. 2). Specifically, the determined antheridium cells (Fig. 2A-N, highlighted by white dashed circles), primarily located at the basal part of the male, continued their differentiation program. By 78 h (Fig. 2N), a few differentiated antheridia had matured and ruptured, releasing motile sperm (indicated by white arrowheads) into the environment. At the conclusion of the live imaging experiment, a fully developed, cordate hermaphrodite with the newly formed meristem notch and archegonia was observed (Fig. 2U,V), still connected to the original male body, marking the completion of the male-to-hermaphrodite transition and the *de novo* formation of a new meristem. Multiple independent male samples exhibited comparable patterns of conversion and new meristem formation during the time-lapse imaging experiments (Fig. 2, Figs S4 and S5).

### Dynamic lineage alterations and single cell-derived *de novo* meristem formation

**Fig. 3.**
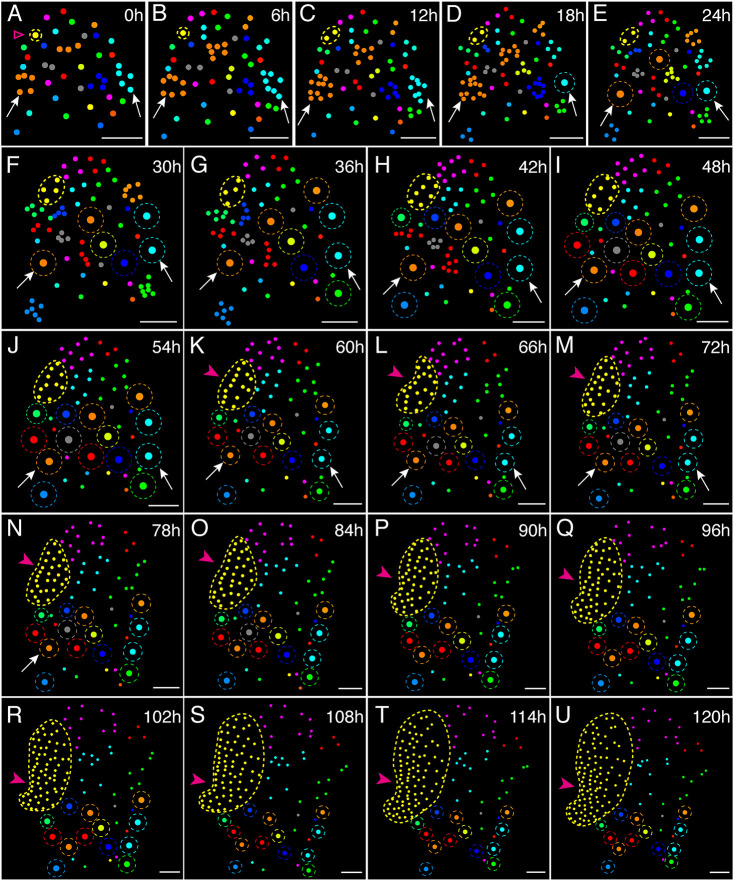
**Cell lineage dynamics during the male-to-hermaphrodite conversion in *Ceratopteris*.** (A-U) Nuclei in the confocal images of Fig. 2A-U (Sample 1) were segmented and labeled with unique IDs. Each dot represents the location of an individual nucleus in the confocal images. Each dashed outline in D-U indicates the location of an individual mature antheridium. Individual nuclei at 0 h are labeled with different colors as a reference for the lineage analysis, and the same color is assigned to progeny cells derived from the same cell in images taken at 6-120 h. (A) The magenta open arrowhead at 0 h indicates the meristem progenitor cell (MPC, colored in yellow) of the lineage contributing to the newly formed meristem. (A-U) Yellow dashed outlines indicate the MPC lineage. (K-U) Magenta arrowheads indicate *de novo* formation of a multicellular meristem. (A-N) White arrows indicate the two representative antheridia also shown in Fig. 2A-N. Scale bars: 50 μm. Three independent samples were analyzed, showing comparable results. Results of lineage dynamics for Sample 2 and Sample 3 are included in Figs S6 and S7, respectively.

To quantitatively assess cellular dynamics during the male-to-hermaphrodite conversion, we used an established computational pipeline (Geng et al., 2022) and performed two-dimensional (2D) image analyses on the time-lapse imaging data. In each image, every nucleus identified by the GFP signal was segmented and assigned a unique ID, with its position represented by a solid circle. Using these segmented nuclei, we constructed dynamic lineage maps at various time points (Fig. 3, Figs S6 and S7). Specifically, at the initial time point (0 h, Fig. 3A, Figs S6A and S7A), each non-antheridium cell in the male was designated as the progenitor of a lineage across subsequent time points, with adjacent nuclei labeled in different colors to visualize lineage development over time. With samples captured every 6 h, our live imaging approach enabled us to observe each cell division throughout the conversion process (Fig. 2, Figs S4 and S5). Lineage tracking was performed based on observed divisions, and cells (nuclei) belonging to the same lineage were consistently labeled with the same color from the first to the last time point (Fig. 3A-U, Figs S6A-U and S7A-W). In addition, at 0 h, nuclei within the same antheridium were grouped and labeled with the same colors. Subsequently, at later time points, nuclei from the same mature antheridium were grouped into a large dot, with the antheridium outlined by a dashed circle. Both the large dot and dashed circle were colored the same as their ancestor cell(s) from the initial image at 0 h (Fig. 3A-U, Figs S6A-U and S7A-W).

Among all the color-coded lineages (Fig. 3A-U, Figs S6A-U and S7A-W), a single lineage – labeled in yellow – consistently expanded, increasing in cell number and forming the largest sector (highlighted with yellow dashed circles in Fig. 3, Figs S6 and S7). Remarkably, this lineage exclusively contributed to the cells that formed the newly formed meristem (Fig. 3U, Figs S6U and S7W, indicated by magenta arrowheads). In contrast, none of other lineages produced cells that became part of the new meristem (Fig. 3U, Figs S6U and S7W). The progenitor of this yellow sector (Fig. 3U, Figs S6U and S7W) was traced back to a single non-antheridium cell at the initial time point (indicated by open magenta arrows in Fig. 3A, Figs S6A and S7A), which we refer to as the meristem progenitor cell (MPC) during the male-to-hermaphrodite conversion.

### The single MPC lineage undergoes two main phases of cell proliferation

**Fig. 4.**
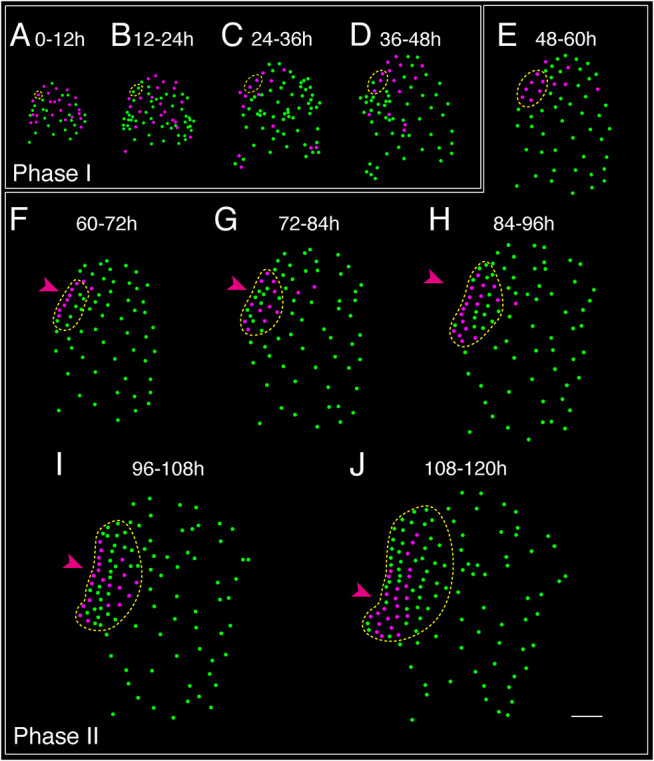
**Cell division dynamics during the male-to-hermaphrodite conversion.** Each colored dot indicates the nucleus of confocal images (Sample 1), except for mature antheridia, which were not included in division quantification. (A-J) Magenta dots indicate cells that underwent division; green dots indicate cells that remained undivided during the indicated 12 h period. (F-J) The magenta arrowheads indicate the initiation and development of a multicellular meristem. The yellow dashed outlines indicate the MPC lineage. Scale bar: 50 μm. Three independent samples were analyzed, showing comparable results. Results of cell division dynamics for Sample 2 and Sample 3 are included in Figs S8 and S9, respectively. Phase I and Phase II indicate the two main developmental stages during the male-to-hermaphrodite conversion.

The lineage atlas (Fig. 3, Figs S6 and S7) revealed that the developmental transition from males to hermaphrodites, as well as the *de novo* formation of a new meristem, was closely associated with active cell divisions. To further explore the dynamics of cell division at both spatial and temporal resolutions, we generated two-color-coded division maps for all of the cells potentially involved in the male-to-hermaphrodite conversion, with a focus on non-antheridium cells. Mature or established antheridia were excluded from this analysis. Based on divisions observed in the time-lapse images (Fig. 2, Figs S4 and S5), we projected cells that underwent division (colored magenta) or remained undivided (colored green) during 12 h time frames (0-12 h, 12-24 h, etc.) onto the segmented images (Fig. 4A-J, Figs S8A-J and S9A-K). The spatial division maps of the samples (Fig. 4, Figs S8 and S9) showed a clear and conserved pattern of division activity, which can be categorized into two distinct phases. Phase I represented the early stage of the male-to-hermaphrodite conversion, occurring prior to the initiation of new meristems (Fig. 4A-D, Figs S8A-D and S9A-E). Typically, this phase occurred within the first 48-60 h after exposure to an antheridiogen-free environment (Fig. 4A-D, Figs S8A-D and S9A-E). During Phase I, multiple non-antheridium cells underwent division within each 12 h time frame, with division locations and patterns appearing largely random (Fig. 4A-D, Figs S8A-D and S9A-E). Phase II, on the other hand, represented the later stage of the conversion, corresponding to the period from new meristem initiation to establishment (Fig. 4E-J, Figs S8E-J and S9F-K). During this phase, cell divisions became progressively restricted to the newly formed meristems (within the MPC lineage, yellow dashed circles), reflecting the active proliferation occurring within these structures and driving their further development (Fig. 4E-J, Figs S8E-J and S9F-K).

**Fig. 5.**
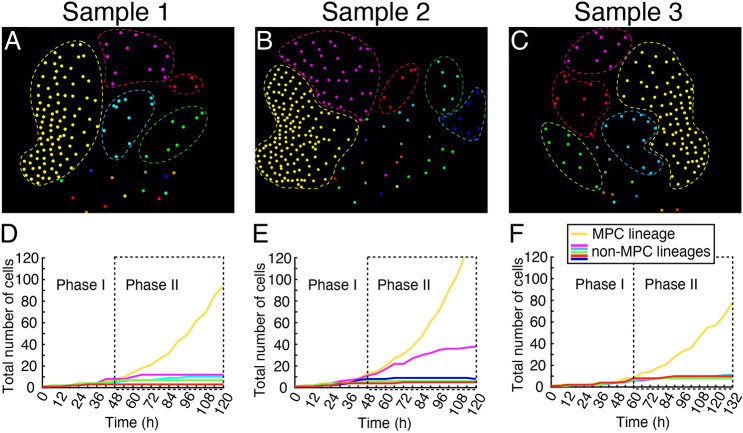
**Quantitative analysis of cell number and division for multiple lineages during the male-to-hermaphrodite conversion.** (A-C) Lineage maps of three samples (Samples 1-3) at the last time point of live-imaging. Each colored dot represents the nucleus in confocal images, except for mature antheridia. Nuclei from the same lineage are labeled in the same color. The five major cell lineages contributing to new hermaphrodite formation are highlighted with dashed outlines for each lineage map. (D-F) Total cell numbers for each of the five lineages at indicated time points during the entire live-imaging period. Phase I and Phase II indicate the developmental stages during the male-to-hermaphrodite conversion.

From the cell lineage maps, we identified multiple lineages, including the MPC lineage (Fig. 5A-C, highlighted by dashed circles in various colors), which originated from males and were involved in cell proliferation and the male-to-hermaphrodite conversion. Subsequently, we performed quantitative analyses of the developmental trajectory, total cell count, and division events for each lineage at different time points from each independent sample (Sample 1: Fig. 5A,D; Sample 2: Fig. 5B,E; Sample 3: Fig. 5C,F, Tables S1-S2). Remarkably, only the MPC lineage (labeled in yellow) in each sample exhibited dominant proliferation activity compared to other identified lineages of non-antheridium cells (Fig. 5A-C). At the conclusion of the live imaging, the MPC lineage contained considerably more cells than any other lineage. We quantified the total number of cells for each lineage at different time points (Fig. 5D-F, Table S1) and the division events from these lineages during each 12 h time frame (Table S2). During Phase I, all five lineages showed largely comparable cell numbers and division events (Fig. 5D-F, Tables S1-S2). However, starting from the early time points in Phase II, the MPC lineage showed high division activity, with a rapid increase in cell number over time (Fig. 5D-F, Table S2). Meanwhile, the other lineages (magenta, red, blue, or green) did not contribute cells to the meristems, showed reduced division activity during Phase II, and eventually became mitotically inactive (Fig. 5D-F). Taken together, both spatial lineage tracking (Fig. 3, Figs S6 and S7) and temporal quantifications of cell number and division (Fig. 5, Table S2) demonstrated that only the MPC lineage contributes to the *de novo* formation of new meristems, characterized by high division activity during Phase II.

### Division orientation of MPC impacts progeny division activity

**Fig. 6.**
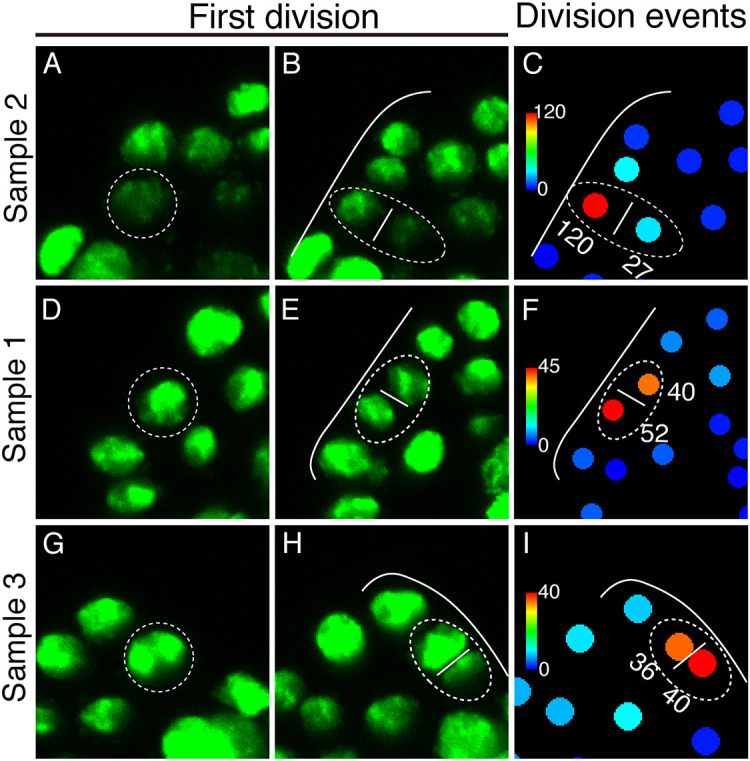
**Division patterns of the meristem progenitor cell during the male-to-hermaphrodite conversion.** (A,B) The first division of the meristem progenitor cell (MPC) in Sample 2 occurred in a periclinal orientation. (C) Total division events for each cell and its progenies over the entire imaging period after the first division of the MPC (6-120 h) are quantitatively indicated by color, with the scale from blue (0) to red (120). (D,E) The first division of the MPC in Sample 1 occurred in an anticlinal orientation. (F) Total division events for each cell and its progenies over the entire imaging period after the first division of the MPC (12-120 h) are quantitatively indicated by color, with the scale from blue (0) to red (45). (G,H) The first division of the MPC in Sample 3 occurred an anticlinal orientation. (I) Total division events for each cell and its progenies over the entire imaging period after the first division of the MPC (6-132 h) were quantitively indicated by color, with the scale from blue (0) to red (40). (A-I) White dashed outlines indicate the first division of the MPC in each sample. (B,C,E,F,H,I) Solid white lines indicate the outlines of gametophytes.

We then focused on the MPC lineage and examined the behavior of the MPC and its daughter cells. The initial division of the MPC appeared to occur in a flexible and unpredictable orientation, with both periclinal and anticlinal orientations observed in our samples (Fig. 6). However, the orientation of this initial division influences the subsequent division activity with the MPC lineage. Specifically, in Sample 2, the initial periclinal division of the MPC (Fig. 6A-C) resulted in one daughter cell remaining in the outermost/marginal layer, while the other daughter cell was positioned in the second, inner layer. Following this division, the progeny of the outermost daughter cell increased to 120 cells, whereas the inner daughter cell produced only 27 cells (Fig. 6C, yellow lineage from B-U in Fig. S6). In contrast, in Samples 1 and 3, the initial division in the anticlinal orientation resulted in both daughter cells remaining in the outermost layer (Fig. 6D-I). Consequently, the progeny of the two daughter cells exhibited comparable division events (52 versus 40 cells in Sample 1; 40 versus 36 cells in Sample 3) (Fig. 6F,I). These findings suggest that the orientation of the MPC division determines the position of its daughter cells, which in turn influences their division activity during meristem development.

### Positional signal drives *de novo* meristem development

**Fig. 7.**
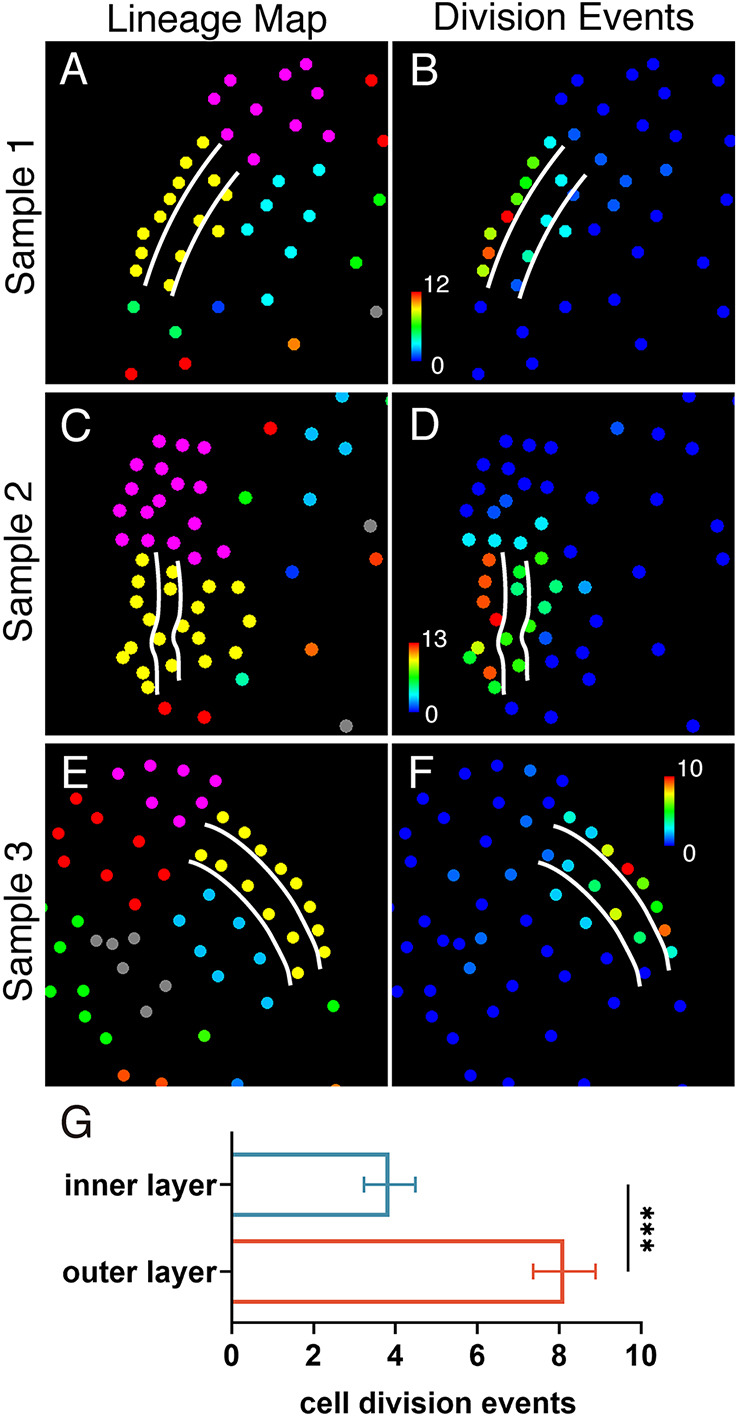
**Layer-specific cell division activity during the *de novo* formation of meristems in *Ceratopteris*.** (A-F) Cell lineages (A,C,E) and quantitative division maps (B,D,F) for Sample 1 (A,B), Sample 2 (C,D), and Sample 3 (E,F) at the starting point of meristem notch formation (60 h for Sample 1, 60 h for Sample 2, and 72 h for Sample 3). (B,D,F) Total division events for each cell and its progeny during the following 60 h period in each sample are shown using RGB color scales: blue (0 divisions) to red (12 divisions) in B, blue (0) to red (13) in D, and blue (0) to red (10) in F. (A-F) White lines highlight the outermost (first) and inner (second) layers of the *de novo* formed multicellular meristems. (G) Average division events for cells located in the outermost layer (*n*=24 cells from the three samples) or the inner layer (*n*=14 cells from the three samples) during the 60 h period from the initiation to the establishment of a meristem notch. ***P<0.0001 (two-tailed Welch's *t*-test).

Our findings led us to propose a hypothesis that the marginal layer plays a crucial role in driving the *de novo* formation of new meristems. To test this hypothesis, we performed a comprehensive analysis of cell division events spanning from the initiation to the establishment of new meristems (Fig. 7). Based on the lineage atlas (Fig. 3, Figs S6 and S7), we identified each cell within the MPC lineage (colored yellow) at the stage when new meristem initiation became apparent (Fig. 7A,C,E). Next, for each sample (Fig. 2, Figs S4 and S5), we calculated the total number of divisions of each cell and its progeny, comparing cells from the outermost layer with those from the second (inner) layer over a 60 h period (Fig. 7A-F, Table S3). We then generated color-coded division maps and projected the division counts to each cell at the first time point of the analyzed frame (Fig. 7B,D,F), quantitatively representing division activity with a color scale from high (red) to low (blue) (Fig. 7B,D,F). This analysis revealed that cells from the marginal layer of the newly formed meristems exhibited higher division activity compared to those from the inner layer. Specifically, the former had an average of 8.1 division events, which was significantly higher than the 3.9 division events observed in the latter (Fig. 7G). These findings demonstrate that the marginal layer of the *de novo* formed meristems, originating from the MPC, drives meristem formation during the male-to-hermaphrodite conversion.

### Mathematical model suggests plausible mechanisms underlying MPC establishment

**Fig. 8.**
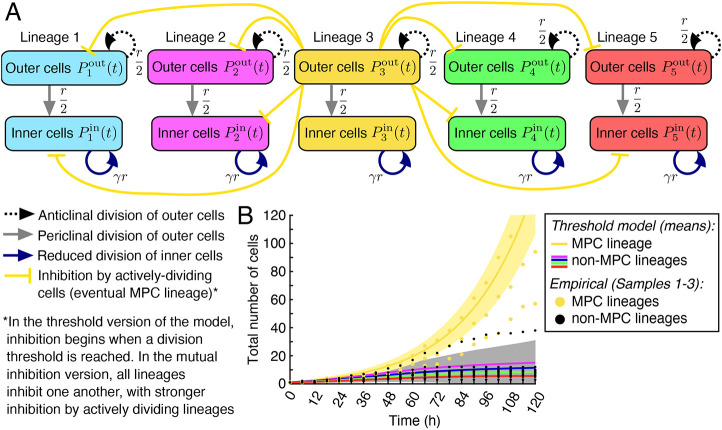
**Mathematical modeling of lineage dynamics at the population scale.** (A) A simple, population-level mathematical model was used to simulate the number of inner cells and outer (marginal) cells in each of five lineages in time. The model assumes all cell lineages are initially identical and undergo stochastic division. Fluctuations in division timing may then lead to one lineage having slightly more cells; when a threshold is reached, the marginal cells in the most actively dividing lineage inhibit the growth of other lineages. This very simple model illustrates candidate mechanisms that may underlie MPC establishment and suggests directions for future work. (B) The stochastic model was simulated 1000 times and the mean cell numbers were computed in time. Specifically, in a post-processing step, the five cell-number curves for each simulation were sorted based on their final number of cells at 120 h and then averaged within each group. (For example, the magenta curve represents the mean trajectory for the second largest lineage in each simulation, averaged across 1000 simulations.) The shaded yellow region indicates ±1 s.d. Rather than showing the s.d. for each non-MPC lineage, the region in which the associated plus (or minus) standard deviation curves are found is shaded in gray. (B) The gold points are the empirical cell numbers for the MPC lineage reproduced from Fig. 5D-F; the black points are the empirical cell numbers for all of the other lineages in Fig. 5D-F. See Figs S10-S12 for additional example simulations.

Based on our data in Figs 5 and 7, we developed a simple mathematical model to simulate the division dynamics of five cell populations, each associated with one lineage generated by its own single marginal cell at 0 h (Fig. 8A). We further sub-divided these five cell populations into inner cells and outer (marginal) cells, and our non-spatial model tracks the number of inner and outer cells in each lineage in time. Our model relies on four main rules: first, supported by our empirical results in Table S4, we specified that anticlinal and periclinal division are equally likely. Second, following our data in Fig. 7G, we set the division rate of inner cells to be half that of marginal cells. Third, we let all cells undergo stochastic division, initially with the same rate. This models the Phase I dynamics in Figs 3 and 4, with division occurring seemingly randomly across the gametophyte. Fourth, we took into account previous experiments that showed that, when actively dividing meristem cells were ablated, cells outside the original meristem increased or regained division activity for cell proliferation (Geng et al., 2022; Withers et al., 2023). In contrast, ablation of mitotically inactive cells had no detectable effect on division activity in other cells (Geng et al., 2022). Based on these observations, we considered two closely related approaches for incorporating inhibition into our model. In our ‘threshold version’, we specified that, when one lineage reaches a cell count that surpasses the cell counts in all other lineages by a certain threshold, the marginal cells in that ‘leading’ lineage inhibit the division activity of all of the other lineages. This threshold can be reached due to stochastic variation in the timing of cell divisions. In our ‘mutual inhibition version’, we removed dependence on a threshold and instead assumed all lineages inhibit one another, with inhibitory signals produced by marginal cells. Stochastic variation can again lead to one lineage exhibiting higher division activity, which is then amplified by the increased inhibitory signals that it produces. See Materials and Methods for full details of our mathematical model and GitLab for our publicly available code (https://gitlab.com/alexandriavolkening/population-model-for-meristem-dynamics-in-ferns).

We present results based on the threshold version of our model in the main text, and explain the non-threshold version in the supplementary Materials and Methods; the two versions of our model produce qualitatively similar behavior under our baseline parameters. As shown in Fig. 8B, our simple model is able to reproduce cell-count trajectories and phases of cell behavior similar to those observed empirically. In Fig. 8B, Figs S10, S11 and S12E,F, we plotted the curves with colors based on their final number of cells, with the largest population – essentially the MPC lineage – plotted in yellow. We tested how these results depend on various model parameters in Fig. S12. Notably, our model does not *a priori* specify that one cell is the MPC at the beginning of the simulation. Instead, a MPC lineage emerges from a pool of initially identical cells in our simulations simply as a result of stochastic division and a relatively strong inhibitory signal triggered by high division activity in marginal cells.

### Male-to-hermaphrodite conversion and MPC establishment depend on new DNA synthesis and cell cycle progression

**Fig. 9.**
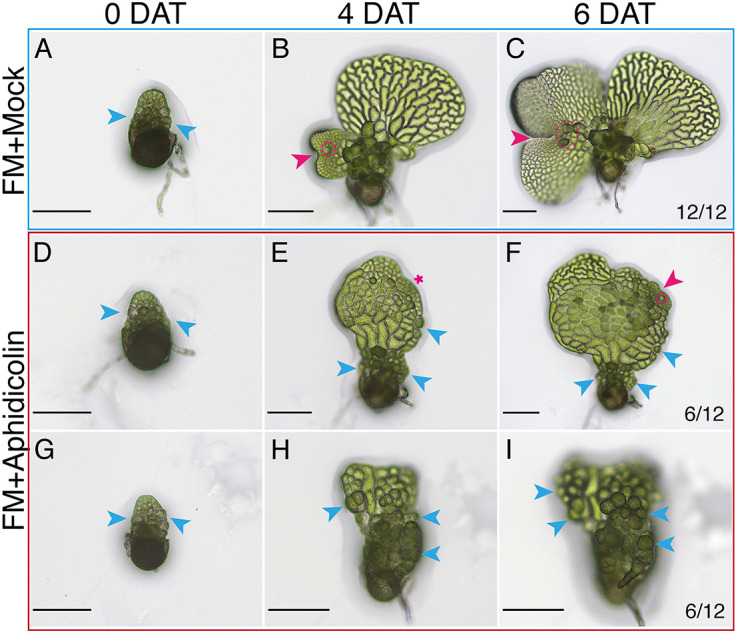
**Aphidicolin treatment disturbs the conversion of males to hermaphrodites in *Ceratopteris*.** (A-I) Males (at 2 DAG) were transferred from CFM to FM with either a mock treatment (A) or 30 μg/ml Aphidicolin (D,G). Time-lapse light micrographs were then taken at the indicated days after treatment (DAT). (A-C) In the absence of antheridiogen and with a mock treatment, the male successfully converted into a new hermaphrodite at 6 DAT. (D-I) In the absence of antheridiogen but in the presence of Aphidicolin, males either partially converted into hermaphrodites with reduced and disturbed meristems (D-F, 6 out 12) or remained as males (G-I, 6 out 12) at 6 DAT. (B,C,F) Magenta arrowheads indicate the *de novo* formation of meristems; (B,C,F) magenta dashed outlines highlight archegonia on the newly formed hermaphrodites. (A,D-I) Blue arrowheads indicate several representative antheridia. (E) The magenta asterisk shows the site of active proliferation during the conversion. Scale bars: 50 μm.

Our data demonstrate a close association between cell division activity and the conversion from males to hermaphrodites. To assess the impact of cell cycle progression on this process and MPC establishment, we developed an assay to systematically evaluate the effects of Aphidicolin, an inhibitor of DNA polymerases α and δ, which blocks the S phase of the cell cycle (Planchais et al., 2000; Ishikawa et al., 2011). As revealed in the micrographs, when transferred to antheridiogen-free FM with the mock control (Fig. 9A), the representative male (12 out of 12) quickly converted to a hermaphrodite, exhibiting a newly formed meristem (Fig. 9B, magenta arrowhead) and an archegonium (Fig. 9B, magenta dashed circle) at 4 days after treatment (DAT) (Fig. 9B). This newly formed meristem continued to proliferate, resulting in a heart-shaped prothallus with a fully established notch (Fig. 9C, magenta arrowhead) and several archegonia (Fig. 9C, magenta circle) at 6 DAT (Fig. 9C). In contrast, when transferred to antheridiogen-free FM with Aphidicolin, the conversion of males to hermaphrodites was disrupted (Fig. 9D-F) or halted (Fig. 9G-I) during the time window (0-6 DAT) we examined. Among the 12 male samples in the experiment, half (6/12) maintained male characteristics and continued to produce antheridia (Fig. 9G-I). At 6 DAT, the majority of cells within these males differentiated into sperm-producing antheridia (Fig. 9I). The other half (6/12) exhibited reduced or disturbed conversion to hermaphrodites (Fig. 9D-F). At 4 DAT, one representative male initiated a new proliferation site (indicated by the magenta asterisk in Fig. 9E), which gave rise to a meristem and an archegonium at 6 DAT (indicated by the magenta arrowhead and the dashed circle, respectively, in Fig. 9F). However, at 6 DAT, the newly formed meristem was small and exhibited abnormal morphology, and the overall size of the prothallus was considerably smaller compared to the mock control (Fig. 9C,F).

**Fig. 10.**
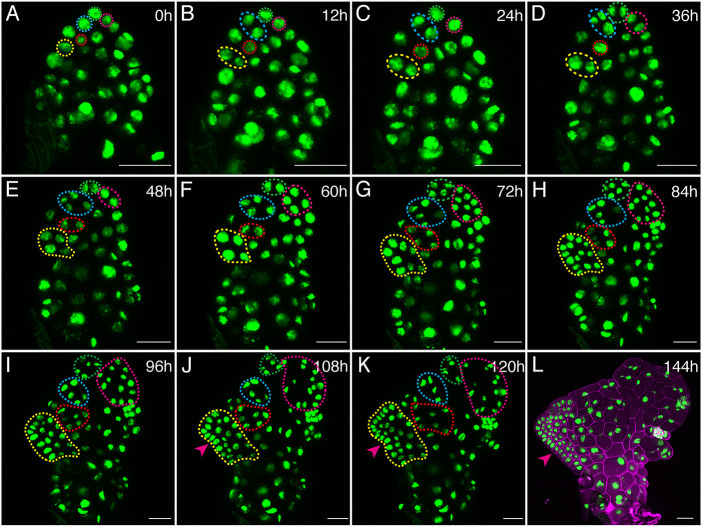
**Time-lapse confocal imaging reveals disturbed male-to-hermaphrodite conversion in the presence of Aphidicolin.** (A-L) *Z*-projection views of GFP signals (green) were taken from a male gametophyte expressing the *pCrUBQ10::H2B-GFP::3′CrUBQ10* transgenic reporter (Sample 1), treated with Aphidicolin. (A) The male (at 2 DAG) was transferred from CFM to FM supplemented with 30 μg/ml Aphidicolin and immediately imaged by laser confocal microscopy (0 h). (B-L) The gametophyte was live-imaged every 12 h up to 144 h when the male converted to a hermaphrodite. (A-K) Color-coded (yellow, blue, red, green, and magenta) dashed outlines highlight different non-antheridium lineages. (J-L) Magenta arrowheads indicate the newly formed meristem. (L) Merged GFP (green) and PI (magenta) channels. Scale bars: 50 μm. At least four samples were live-imaged with the same settings and time intervals, showing comparable results. See Fig. 11A-D for corresponding quantitative analysis of cell counts.

Next, we performed long-term time-lapse confocal imaging of male gametophytes treated with Aphidicolin. Transgenic spores (*pCrUBQ10::H2B-GFP::3′CrUBQ10*) were initially inoculated onto growth medium supplemented with antheridiogen. At 2 DAG, males were transferred to antheridiogen-free growth medium with either the mock control (Fig. S13) or Aphidicolin (Fig. 10). Following the transfer, we performed live imaging of these gametophytes every 12 h (Fig. 10A-L and S13A-L), using the same procedure described earlier. The time-lapse images (Fig. 10A-L) revealed that, compared with the mock control (Fig. S13) or samples cultured under normal conditions (Fig. 2, Figs S4 and S5), Aphidicolin-treated samples exhibited slow division activity, with disrupted meristem initiation and proliferation (Fig. 10A-L). For example, in one representative sample, the initiation of a new meristem (Fig. 10J,K, arrowheads) was not evident until 108 h after the treatment (Fig. 10J,K). By 120 h, only a limited number of cells had formed in the MPC lineage (Fig. 10K), and even at 144 h, the gametophyte developed only a disrupted meristem (indicated by the magenta arrowhead), lacking a notch (Fig. 10L).

**Fig. 11.**
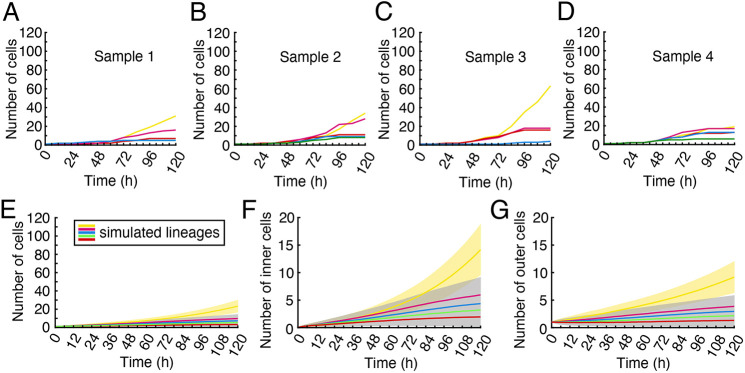
**Quantitative analysis of cell number for multiple lineages in the presence of Aphidicolin and simulated lineage dynamics under reduced division activity.** (A-D) Total cell numbers for each analyzed lineage in Fig. 10 from four independent samples, including Sample 1 (A), Sample 2 (B), Sample 3 (C), and Sample 4 (D), at indicated time points during the entire live-imaging period. Different colors represent different lineages from each sample (A-D), and the sample lineages in Fig. 10A-K and in A are presented with the same colors (yellow, red, blue, green, and magenta). The *y* limits in A-D are the same as in Fig. 5D-F for easier comparison with the untreated populations. (E-G) A simple mathematical model (Fig. 8A) is able to produce qualitatively similar cell number dynamics to the empirical results (A-D) under Aphidicolin treatment, producing fewer total cells (E), both for inner cells (F) and outer cells (G). The results (E-G) are based on simulating the stochastic model 1000 times (threshold version) with the division rate *r* reduced by half for all of the lineages (see Fig. 8A); the resulting lineages were then sorted by their population size at the final time (120 h) for each simulation and averaged across simulations. The yellow shaded region indicates ±1 s.d. for the largest lineage; the gray shaded area indicates the region occupied by ±1 s.d. curves for all of the other lineages. See Fig. S11 for associated simulations of the non-threshold version of the model.

We then traced the divisions of non-antheridium cells and their progeny in the Aphidicolin-treated samples over time, identifying multiple independent cell lineages (Fig. 10A-K, highlighted by dashed circles in various colors), including the potential MPC lineage (yellow circles in Fig. 10A-K). Similar to the quantitative analysis of samples grown on antheridiogen-free FM without Aphidicolin (Fig. 5D-F), we counted the total cell numbers in each cell lineage at different time points from four independent samples grown on antheridiogen-free FM with Aphidicolin treatment (Fig. 11A-D). Unlike the untreated samples (Fig. 5D-F), the MPC lineage in each of the four Aphidicolin-treated samples showed a greatly reduced cell number, with only three or four cells present at 48 h (Fig. 11A-D). At 120 h, the MPC lineage also showed generally lower total cell numbers, ranging from 19 to 63 (Fig. 11A-D). More importantly, in two samples (Fig. 11B,D), the MPC lineage was indistinguishable from the other lineages based on viewing the cell counts. In the other two samples (Fig. 11A,C), the differences in cell number between the MPC lineage and other lineages were also smaller compared to those in the untreated samples (Fig. 5D-F).

In parallel with this work, our mathematical model provides a simple and independent method to test the impact of disturbed cell cycle progression during meristem establishment. We simulated Aphidicolin treatment by reducing the baseline division rate (*r* in Fig. 8A) of all lineages by half (Fig. 11E-G). This simulated perturbation produced qualitatively similar cell-number trajectories *in silico*, making the candidate MPC lineage less distinguishable from the other lineages. Comparing Fig. 11E-G and Fig. S11B, we observe that both versions of our model behave similarly under reduced division activity. Taken together, our empirical observations and model simulations suggest that re-entering and maintaining cell cycle progression are essential for the male-to-hermaphrodite conversion and the establishment of the MPC lineage.

## DISCUSSION

### Formation of a single cell-derived multicellular meristem during sex conversion

In this study, we examined the *de novo* induction of new meristems in *Ceratopteris* during the conversion from males to hermaphrodites, a naturally occurring and crucial process induced by the absence of antheridiogen (Hickok et al., 1987; Eberle and Banks, 1996; Cheruiyot and Schwartz, 2007). Using a combination of time-lapse confocal imaging of the transgenic reporter, drug treatment, quantitative image analyses, and mathematical modeling, we constructed a cell division trajectory and lineage atlas for this process.

In the time-lapse experiments, gametophytes were imaged directly on their Petri dishes containing growth medium, without any mounting or dissection. After imaging, the samples, along with their Petri dishes, were promptly returned to the growth chamber adjacent to the confocal microscope and cultured under identical conditions until the next imaging time point. As demonstrated in our previous study (Geng et al., 2022), imaging at 6 h intervals effectively captured all division events in gametophytes, enabling comprehensive cell lineage analysis. Repeated observations from both previous (Geng et al., 2022) and current studies confirm that this imaging procedure does not interfere with normal division, growth, or differentiation of gametophytes. First, all live-imaged hermaphrodites developed fully expanded prothalli with distinct meristem notches and adjacent archegonia by the end of long-term live imaging experiments (Geng et al., 2022). Second, high cell division activity was consistently observed during the later stages of imaging (e.g. the yellow MPC lineages after 72 h in the three samples detailed in Table S2), indicating that repeated imaging neither reduced cell division activity nor disrupted normal developmental processes.

Quantitative analysis of time-lapse imaging data has enabled us to identify the cellular behaviors driving the male-to-hermaphrodite conversion, and to compare these with the processes associated with normal male and hermaphrodite development. Male gametophytes lack a meristem (Banks, 1999; Hickok et al., 1987), and in the presence of antheridiogen, most or all cells in a male gametophyte ultimately differentiate into sperm-producing antheridia (Fig. 1H) (Banks, 1999). In contrast, in the absence of antheridiogen, we identified a single non-antheridium cell (Figs 2 and 3, Fig. S14) within the male body that functions as the MPC. The MPC lineage sustains active cell division and exclusively contributes to the formation of the entire new meristem (Fig. 3). At a later stage of the sex conversion, the MPC lineage exhibits an accelerated proliferation rate, while other non-antheridium cell lineages lose division activity and become mitotically inactive (Fig. 4). Previous live-imaging studies beginning at 5 days after spore inoculation (5 DAI) identified two or three marginal cells that exclusively contribute to meristem formation in hermaphrodites (Geng et al., 2022). Future analysis performed earlier than 5 DAI would help determine whether these two or three marginal cells originate from a single MPC, similar to the MPC identified during the male-to-hermaphrodite conversion. Additionally, the spatial and temporal dynamics of cell divisions observed during *de novo* meristem formation suggest two distinct phases: Phase I and Phase II (Fig. 4). These phases appear comparable to the three developmental phases – initiation, transition, and maturation – previously identified during normal meristem development in hermaphrodites (Geng et al., 2022). Specifically, Phase I aligns with the initiation phase of normal meristem development, while Phase II encompasses both the transition and maturation phases categorized in the earlier study (Geng et al., 2022).

Treatment with a cell cycle and DNA synthesis inhibitor greatly disrupted or significantly delayed sex conversion and MPC lineage establishment (Figs 9 and 10), suggesting that re-entering and maintaining cell cycle progression defines an essential step in this process. The *de novo* meristem formation in *Ceratopteris* gametophytes appears to share conserved features with the *de novo* induction of shoot meristems from callus, initiation of axillary meristems, and lateral root primordium development in angiosperm sporophytes (such as Arabidopsis). All these processes require non-meristem cells to regain meristematic identity and re-enter cell cycle progression (Smith and Hake, 1992; Gordon et al., 2007; von Wangenheim et al., 2016; Nicolas and Laufs, 2022). Interestingly, these processes in angiosperm sporophytes involve a small group of progenitors rather than a single cell, suggesting a difference in meristem initiation between angiosperms and ferns.

### Division orientation and marginal layer signals dictate division activity

Time-lapse imaging and quantitative analysis demonstrate that positional cues are essential for sustaining active division for the *de novo* induction and subsequent development of new meristems during the conversion. The orientation of the initial division in the MPC determines the division activity of its progeny (Fig. 6). Specifically, an anticlinal division in the MPC results in daughter cells in the marginal layer, both of which maintain high division activity. Conversely, a periclinal division in the MPC leads to the daughter cell in the outermost layer having higher division activity than the other daughter cell that falls in the inner layer (Fig. 6). Positional signals also drive cell proliferation during the establishment of new meristems (Fig. 7). In meristems initiated from the male body, cells in the marginal/outermost layer exhibit significantly higher division activity than those in the inner layer (Fig. 7). This finding aligns with our previous observation in the established meristems of *Ceratopteris* hermaphrodites, where marginal cells show significantly higher division activity than inner cells from the same lineages (Geng et al., 2022). Our mathematical model further supports this observation, as we highlight in Fig. S12B that the difference between the mean *in silico* and *in vivo* cell number trajectories increases as the difference in division activity between marginal and inner cells decreases. Collectively, these findings demonstrate that cell proliferation in newly formed meristems originating from males and in established meristems in hermaphrodites share conserved regulatory mechanisms, both induced by signals from the marginal layer. Interestingly, the *de novo* induction of new meristems and the maintenance of apical meristems in angiosperm sporophytes also involve signals from the epidermis (Nicolas and Laufs, 2022; Gruel et al., 2016; Han et al., 2020; Geng and Zhou, 2021; Han and Zhou, 2022; Knauer et al., 2013). For example, microRNA394, which moves from the epidermis of *Arabidopsis* SAMs to inner cell layers, maintains stem cell functions (Knauer et al., 2013), while microRNA171, which is synthesized in the epidermis, acts as a short-range mobile signal regulating HAM location patterns and the initiation and maintenance of *Arabidopsis* shoot meristems (Han et al., 2020; Geng et al., 2024b). Identifying mobile signals from the outermost layer of *Ceratopteris* gametophytes in future research will provide insights into these processes. Additionally, recent studies have shown that auxin treatments disrupt the organization of the meristematic notch in *Ceratopteris* gametophytes, suggesting a role for auxin signaling in meristem development (Woudenberg et al., 2024). Investigating whether phytohormone signals play a conserved role in shaping diversified meristems across lineages at different stages of the life cycle will be valuable and important.

### Mathematical model of plausible mechanisms underlying MPC establishment

**Fig. 12.**
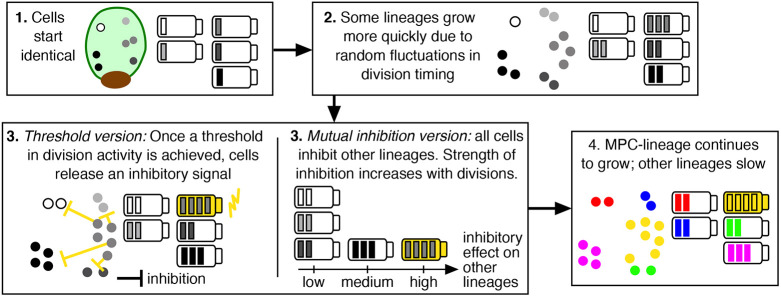
**Summary of the model hypothesis for meristem establishment through selection of a MPC.** The combined empirical findings and mathematical model propose plausible mechanisms underlying meristem cell dynamics during the male-to-hermaphrodite conversion.

Taken together with our empirical findings and previous work (Geng et al., 2022), our simple mathematical model provides a potential explanation for how one cell may go on to outpace other lineages in its division activity, eventually establishing all the daughter cells in the meristem (Fig. 12). Specifically, Geng et al. (2022) demonstrated that ablating the developing meristem notch in *Ceratopteris* triggers new meristem formation in another region of the gametophyte. One explanation for this observation is that meristem cells could produce an inhibitory signal that prevents cells in other lineages from actively dividing and initiating new meristems (Geng et al., 2022). Such a signal could be generated, for example, in actively dividing marginal cells. In the threshold version of our mathematical model, which can be interpreted as modeling molecular switches, we specified that each cell in the marginal layer began undergoing noisy division at 0 h at an identical rate. Stochastic fluctuations in timing then led to one cell lineage exhibiting higher division activity, and, when a threshold was passed, this ‘winning’ lineage had an inhibitory effect on cell division in other lineages. The result of this process was further amplification of the growth of the ‘winning’ lineage relative to others *in silico* (Fig. 12). In the mutual inhibition version of our model, we instead let all lineages inhibit one another, with the strength of inhibition depending on the number of marginal cells in each lineage. Again, stochastic fluctuations led to one lineage exhibiting more division activity, later amplified by its increased inhibitory effect on others.

While our initial model is a simplified representation of complex spatiotemporal dynamics, it confirms that noisy division in initially identical cell populations, coupled with an inhibitory signal dependent on division activity, is sufficient to drive one cell to establish the entire meristem. Although in agreement in terms of this conclusion, the two versions of our model offer slightly different perspectives on the mechanisms behind these overall dynamics. Notably, we found that the threshold and mutual inhibition versions of our model offer qualitatively different predictions under low noise (Fig. S12E,F). Specifically, when the difference in noisy division activity between lineages is low, the threshold in our threshold model is not reached in the timeframe that we consider and instead all of the lineages exhibit MPC-like growth. In contrast, in the mutual inhibition model, low noise leads to all of the lineages behaving like non-MPC lineages. This relationship to low noise highlights a key difference in the two versions of our model.

There are many places to improve and build on our initial modeling study. As an example, in the future it will be useful to further investigate parameter sensitivity and unpack how alternative forms for the inhibitory terms in the model affect predictions. It is also important to note that our model seeks to describe the growth of cell lineages only during the time of empirical data collection. For example, we expect that additional signals are active later on in *Ceratopteris* development to constrain the growth of the MPC lineage to realistic cell numbers. Finally, moving forward, an exciting direction is to incorporate cell division dynamics and inhibitory signals into a spatial model. In the future, we plan to expand this initial model to develop and test spatial models of cell signaling and meristem establishment during gametophyte development.

In summary, the discovery of the single-cell-derived multicellular meristem during the gametophyte phase in *Ceratopteris* provides new insights into the conserved and diversified mechanisms underlying *de novo* meristem formation in land plants. It also presents an efficient system for studying the balance between cell division and differentiation at single-cell resolution in multicellular organisms, through a combination of experimental approaches and mathematical modeling.

## MATERIALS AND METHODS

### Plant materials and growth conditions

Spores of *Ceratopteris richardii* Hn-n were surface-sterilized and spread on Petri dishes containing FM (fern medium) with 0.5×MS salts and vitamins (PhytoTechnology Laboratories) at pH 6.0, and in 0.7% (w/v) agar (Sigma-Aldrich). Individual gametophytes were transferred into each well of the 48-well plate containing FM for self-fertilization and the generation of sporophytes. Calli were induced from the shoot tips and fronds of young sporophytes with the induction media containing 1×MS salts and vitamins, 2% (w/v) sucrose, 1 mg/l benzylaminopurine (BAP) and 0.7% (w/v) agar at pH 5.8. The CFM (conditioned FM) plates containing Antheridiogen, which promotes the male program, were prepared as described previously (Banks et al., 1993; Geng et al., 2024a). Specifically, 0.1 g *Ceratopteris her19* spores were sterilized and cultured in 1000 ml of liquid FM, and shaken at 110 rpm at 29°C in continuous light for 30 days in the growth chamber (Percival). The suspension culture was then filtered to remove gametophytes, and the liquid was recovered to prepare CFM containing 0.5×MS salts and vitamins (pH 6.0), and 0.7% agar. The efficacy of CFM plates for inducing male programming was assayed and confirmed using Hn-n spores before the live-imaging experiments. Gametophytes, young sporophytes, and calli were grown under continuous light at 29°C in the growth chamber (Percival). Fertile sporophytes were grown in Purdue Lilly Greenhouse Facility for spore collection.

### DNA constructs and plant transformation

To generate a fluorescent nuclear marker with high and uniform expression in *Ceratopteris* gametophytes, a *pCrUBQ10::H2B-GFP::3′CrUBQ10* expression cassette was constructed. The *CrUBQ10* gene was initially identified in the *de novo* transcriptome (Geng et al., 2021) and the genomic sequence of *CrUBQ10* was located in the published genome (Marchant et al., 2022). A 3094 bp promoter of *CrUBQ10* was amplified from the *Ceratopteris* genome using the primers (5′-ACAAgcggccgcCTAAGTGTAAAGTCACTGGCACCAAGTG-3′ and 5′-ACAAgcggccgcGTTGGCAGTAGCACGAATAAGAAATCAC-3′) and then cloned into the 5′ end of the previously reported *Histone 2B* (*H2B*)*-GFP* fragment (Geng et al., 2022; Han et al., 2020). An 863 bp 3′ terminator of *CrUBQ10* was amplified from the genome using the primers (5′-TACAggcgcgccGGGTTTGATCTGTGTGCCTGGAG-3′ and 5′-TACAggcgcgccCAAGCTAAACTGCCTACCCAACATGT-3′) and cloned into the 3′ end of the *pCrUBQ10::H2B-GFP* fragment. Then, the *pCrUBQ10::H2B-GFP::3′CrUBQ10* expression cassette was cloned into pMOA34 for stable transformation.

*Ceratopteris* stable transformation was carried out using microparticle bombardment, following the detailed procedure described by Plackett et al. (2014). Plasmid DNA was coated with tungsten and delivered into *Ceratopteris* calli using the Bio-Rad Biolistic PDS-1000/He particle delivery system at 1100 psi, as previously described (Plackett et al., 2014; Geng et al., 2022). Regenerated shoots were individually selected with hygromycin (40 µg/ml). Spores from each transgenic line were harvested, surface-sterilized, and spread on FM containing hygromycin to test hygromycin resistance and confirm stable transformation. The H2B-GFP signal of transgenic gametophytes was examined using a Zeiss LSM880 confocal microscope as described previously (Geng et al., 2022). At least three independent transgenic lines (lines 10, 14, and 18) showed comparable and ubiquitous expression patterns in *Ceratopteris* gametophytes (Figs S2 and S3). Notably, both the previously published *pCrHAM::H2B-GFP::3′CrHAM* reporter (Geng et al., 2022) and the *pCrUBQ10::H2B-GFP::3′CrUBQ10* reporter generated in this study exhibit ubiquitous expression signals in *Ceratopteris* prothalli. However, the *pCrHAM::H2B-GFP::3′CrHAM* reporter is not expressed in the gametes of *Ceratopteris* gametophytes (Geng et al., 2022), whereas the *pCrUBQ10::H2B-GFP::3′CrUBQ10* reporter exhibits bright expression signals in the gametes from both antheridia and archegonia (Fig. 2, Figs S2 and S3), making it particularly well-suited to this study. Additionally, gametophytes of *pCrUBQ10::H2B-GFP::3′CrUBQ10* transgenic lines displayed no developmental defects compared to wild-type controls (Fig. S16). Line 10 was used for further time-lapse imaging and image analysis (as shown in Fig. S2 and Fig. 2). Spores of transgenic *Ceratopteris* are available upon request.

### Confocal live imaging

Transgenic *Ceratopteris* gametophytes were live-imaged using a Zeiss LSM880 upright confocal microscope. Spores of the *pCrUBQ10::H2B-GFP::3′CrUBQ10* reporter transgenic lines were surface sterilized and spread on Petri dishes containing CFM. The CFM plates were cultured in a growth chamber (Percival) under continuous light at 29°C. For time-lapse imaging of male-to-hermaphrodite conversion, male gametophytes at 2 days after germination (2 DAG) were transferred from CFM to FM and imaged using confocal microscopy as the first time point (0 h). Following imaging, the gametophyte plates were returned to the growth chamber adjacent to the confocal microscope under continuous light with an intensity of 27±4 W/m^2^ at 29°C, consistent with conditions reported previously (Banks et al., 1993). Gametophytes were maintained under identical conditions between imaging sessions. Live imaging was performed every 6 h until the *de novo* formation of new meristems and adjacent archegonia. Specifically, the newly formed multicellular meristems in the converted hermaphrodites (Fig. 2V, Figs S4V and S5X) were characterized by distinctive cell morphology and division activity. Multicellular meristems in *Ceratopteris* gametophytes are composed of rectangular cells with small dimensions, where each cell is largely occupied by a nucleus (Fig. 2V, Figs S4V and S5X) and exhibits continuous division activity (Fig. 4, Figs S8 and S9). In contrast, cells outside the meristem are significantly larger, with nuclei occupying only a small portion of the total cell volume (Fig. 2V, Figs S4V and S5X), and these cells lose division activity (Fig. 4, Figs S8 and S9). The morphology and division patterns of cells during the initiation and proliferation of egg-producing archegonia were distinct from those of other cell types in converted hermaphrodites. Zoomed-in confocal images of GFP signals, combined with the DIC channel (showing cell outlines), are presented in Fig. S15 to depict developing archegonia in a converted hermaphrodite. At the last time point, live-imaged gametophytes were stained with propidium iodide (PI) to visualize cell outlines. Gametophytes were stained with PI for 1-2 min and rinsed twice with sterilized water as described previously (Geng et al., 2022).

The imaging settings in ZEN software for operating the LSM880 were described previously in detail (Wu et al., 2022a; Geng et al., 2022, 2024a; Geng and Zhou, 2019). Gametophytes were imaged using a Plan-Apochromat 10×/0.45 objective lens with 1.0 μm scanning intervals. GFP was excited with a 488 nm laser line, and emissions were collected in the 490-544 nm range. The transmission (DIC) channel was also excited with the 488 nm laser line and collected to visualize gametophyte cell outlines. PI was excited with a 561 nm laser line, and emissions were collected in the 595-650 nm range. Maximum-intensity *z*-projection views of confocal stacks from GFP or PI channels were processed using Fiji/ImageJ software and are presented in the figures. Additionally, a single optical section from the DIC channel was processed using Fiji to show cell outlines. When necessary, the brightness of each entire image was adjusted using Fiji/ImageJ for clear visualization of nuclei in gametophytes.

### Light microscope imaging

Spores of Hn-n were sterilized and spread on FM plates. Starting at 2 DAG, seven hermaphrodites and seven male gametophytes were live-imaged at 2, 6, 8 and 15 DAG (Fig. 1). During the imaging period, male gametophytes were continuously exposed to antheridiogen. Separately, nine additional male gametophytes were randomly selected at 2 DAG, and each male was individually transferred to antheridiogen-free FM plates to eliminate the potential effect of antheridiogen (Fig. 1). By 15 DAG, all of these male samples had converted into hermaphrodites (Fig. 1L). Light micrographs were taken using the Olympus CKX53 microscope equipped with a Mlchrome 5 Pro digital camera and processed with Mosaic2.3 software. Representative images of a male gametophyte, a hermaphrodite, and a hermaphrodite converted from a male are shown in Fig. 1.

### Cell division quantification and statistical analysis

The total cell numbers and division events of each cell lineage during the analyzed periods were quantified (Fig. 5; Tables S1 and S2). Five cell lineages in each sample that showed high division activity but were unrelated to antheridium differentiation were included in the analysis (Fig. 5). The analyzed lineages are highlighted with the dashed lines in the lineage maps of each sample (Fig. 5A-C). All the cells/nuclei from the same lineage are labeled with the same color (Fig. 5A-F). The two developmental stages (Phase I and Phase II) are also indicated in the graphs (Fig. 5D-F).

The average division events of the cells located at the outermost layer or from the inner (second) layer were quantified over a 60-h period following meristem initiation (Fig. 6; Table S3). The cells from the outermost layer and the second layer of the newly formed meristems were highlighted in both lineage maps and quantitative division maps (Fig. 6). The anticlinal and periclinal cell division events during the male-to-hermaphrodite conversion were analyzed across three imaged samples (Table S4). Given that the outermost layer is the primary driving force in *de novo* meristem formation, and the division orientation within this layer can be clearly categorized as either anticlinal or periclinal relative to the gametophyte boundary, every cell division event occurring in the outermost layer of the newly formed meristems was recorded for Samples 1-3 throughout the entire imaging period (Table S4). The statistical significance was determined using a two-tailed Welch's *t*-test. All charts were plotted by GraphPad Prism 9.5.1.

### Aphidicolin treatment and live imaging

The effect of Aphidicolin on male-to-hermaphrodite conversion was examined. Wild-type spores were surface sterilized and spread on CFM plates to induce the male developmental program. At 2 DAG, male gametophytes of similar size were randomly selected from the CFM plates and transferred onto FM with either a mock treatment (solvent only, 0 μg/ml Aphidicolin) or 30 μg/ml Aphidicolin. Each sample was cultured individually on separate Petri dishes to avoid any potential effects of antheridiogen released from neighboring gametophytes and maintained under identical growth conditions. The gametophytes were imaged at the indicated days after treatment (Fig. 9) through the Olympus CKX53 microscope.

To investigate the cellular dynamics of *Ceratopteris* gametophytes in response to Aphidicolin, confocal time-lapse imaging was performed. Spores of the *pCrUBQ10::H2B-GFP::3′CrUBQ10* transgenic reporter line were spread on CFM. At 2 DAG, male gametophytes were transferred to FM treated with either a mock solution or 30 μg/ml Aphidicolin and imaged by confocal microscopy as the starting point (0 h). Time-lapse imaging was performed similarly to the above section, except with the 12 h intervals for this experiment. At 144 h, samples were stained with PI, and both PI and GFP signals were separately collected as previously described. Total cell numbers for each analyzed lineage from four independent samples (Aphidicolin-treated Samples 1-4, shown in Fig. 11A-D) at the indicated time points are included in Table S5.

### Mathematical model

A mathematical model was used to simulate the number of inner and outer (marginal) cells in each of five main populations (i.e. lineages) in time (Fig. 8A). The variable 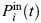 denotes the number of inner cells in the *i*th lineage at time *t*, and 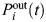 denotes the number of marginal cells in the *i*th lineage at time *t*, where *i*=1, 2, 3, 4 or 5. It is important to stress that the model is a simplified system; it is meant to test the hypothesis that initially identical cell populations can give rise to a large MPC lineage through stochastic fluctuations in division combined with division-induced inhibition. The population size is described as number of ‘cells’ throughout the modeling discussion, but 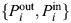 take on non-integer values. Thus, an alternative way to think of 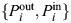 in the toy model is as the concentrations of morphogens that are released by and thus closely related to the number of cells.

Broadly, the mathematical model specifies that cells of all lineages initially undergo stochastic division at a rate *r* for outer cells and a reduced rate *γr* for inner cells. In addition, as shown in Table S4, there is no significant difference in the frequencies of anticlinal and periclinal divisions among the three live-imaged samples, providing experimental evidence for the model assumption. If the number of marginal cells 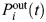 for the *i*th lineage is sufficiently higher than the number of marginal cells in the other lineages, the model assumption is that the marginal cells of the *i*th lineage produce an inhibitory signal that decreases the division activity of all of the other lineages. This is based on ablation experiments (Geng et al., 2022) that suggest meristem cells may inhibit other cells. Through this process, stochastic fluctuations may give rise to one lineage with relatively more marginal cells, and these ‘leading’ marginal cells can go on to sufficiently inhibit all of the other cell populations to emerge as the clear MPC lineage.

Specifically, the model takes the form of a system of stochastic differential equations, as below:

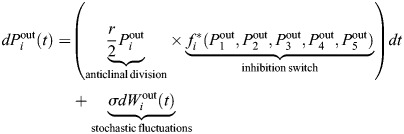
and

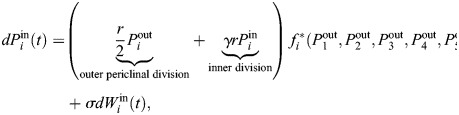
where *r* is the baseline division rate for outer cells; *γ*=0.5 is a parameter that reduces the division rate of inner cells relative to outer cells (determined based on Fig. 7G); 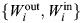 are Wiener processes; *σ* is the noise strength; and 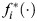 with *∈{thresh, mutual} is a piecewise, Hill-type function that implements inhibition in one of two ways, depending on the version of the model.

In the threshold version of the model, 
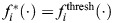
 implements inhibition once a specific threshold is reached in the difference between the number of marginal cells for different lineages. Specifically, it is defined as:

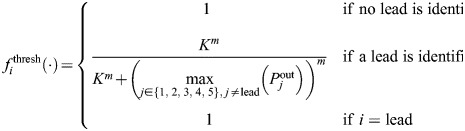
where *K* and *m* are parameters in the Hill-type function term for repressing division of non-lead lineages. [Hill functions (Alon, 2019; Santillán, 2008; Gesztelyi et al., 2012) are used widely in systems biology, including in models of gene regulatory networks and pharmacological dynamics.] The *i*th lineage is defined as the current leading lineage (so that *i*=lead above) according to the following rule:

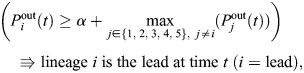
where *α* is a threshold parameter. It is possible for the leading lineage to change in time due to stochastic fluctuations in division activity, but model simulations in Fig. 8B show that eventually one lineage emerges as dominant.

In the mutual inhibition version of the model, 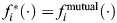 prescribes inhibitory signals from marginal cells to all other lineages according to:

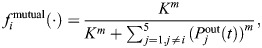
where *K* and *m* take different values in 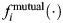 and in 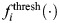. See supplementary Materials and Methods for additional information, including a table of parameters involved in the model and its numerical implementation (Table S6), details on how the model equations were solved and how non-negativity was enforced, a description of the model initial conditions, and an investigation into the effects of the parameters *γ* and *σ* on model results. To further support reproducibility, the model code is publicly available in GitLab (https://gitlab.com/alexandriavolkening/population-model-for-meristem-dynamics-in-ferns).

### Image segmentation and quantitative cell-lineage analysis

The 2D image segmentation was performed as described previously (Geng et al., 2022; Meyer, 1994). Nuclei image segmentation/detection and quantitative lineage analysis were carried out using the Matlab platform. The 2D maximum intensity projection images were processed through three imaging analysis modules, as follows. The first module deals with 2D nuclei segmentation and detection. Initially, the 2D maximum intensity projection images were Gaussian blurred and binarized using Matlab's built-in adaptive thresholding. The binarized images were then processed through distance transformation and subsequently inverted. These processed images underwent built-in watershed segmentation (https://www.mathworks.com/help/images/ref/watershed.html#bup39ap-2) (Meyer, 1994). In cases of over-segmentation, manual corrections were performed using merging and deleting functions, while under-segmentation was corrected using a manual splitting function. As the final step, circles were automatically fitted into the centers of the segmented nuclei for the clear visualization of their positions in the 2D images. The second module generates color map images for the time series of dynamic lineages. Randomly assigned colors were used to label different founding nuclei at the initial time point. Based on manually curated lineage data, daughter nuclei at later time points were identified and colored to match their corresponding founding nuclei. This approach allows the direct visualization and examination of the progression of nuclei lineages over time (as shown in a representative sample in Fig. 3). The third module visualizes statistical results from the quantitative lineage analysis. Using the lineage data, an automatic Matlab script counts the number of cell division events for each nucleus over a specified time period, applying a color scale to represent this quantitative information. Another Matlab script identifies whether nuclei divided during a specified time period, coloring dividing nuclei in magenta and non-dividing nuclei in green.

Notably, mature antheridia form a complex 3D structure containing multiple sperm nuclei (as shown in Fig. 2D-N), which were not included in the segmentation or division quantification in this study. Instead, cells within the same mature antheridium were represented as a single large dot, with the antheridium outlined by a dashed circle. Both the dot and dashed circle were assigned the same color in the lineage maps (Fig. 3, Figs S6 and S7). As part of normal developmental program, mature antheridia eventually rupture and release sperm (Fig. 2). After rupture, the GFP signals from the remaining nuclei in antheridia become weak or undetectable for image analysis. Additionally, GFP signals from released sperm were not included in image analysis or quantification (as shown in Figs 2 and 3).

## Supplementary Material



10.1242/develop.204411_sup1 Supplementary information

Table S1. Total cell number in three samples

Table S2. Cell division events in three samples

Table S3. Cell division events in the outermost and second layers of three samples

Table S4. Anticlinal and periclinal division events in the outermost layer of newly formed meristems across three samples

Table S5. Total cell number in four samples treated with Aphidicolin
