# Supplementary material for: Single cell-derived multicellular meristem: insights into male-to-hermaphrodite conversion and de novo meristem formation in Ceratopteris
Source: Development. 2025 Feb 13;152(3):DEV204411. doi: 10.1242/dev.204411 (PMC11883269; doi:10.1242/dev.204411)
Supplement: Supplementary information [file develop-152-204411-s1.pdf]

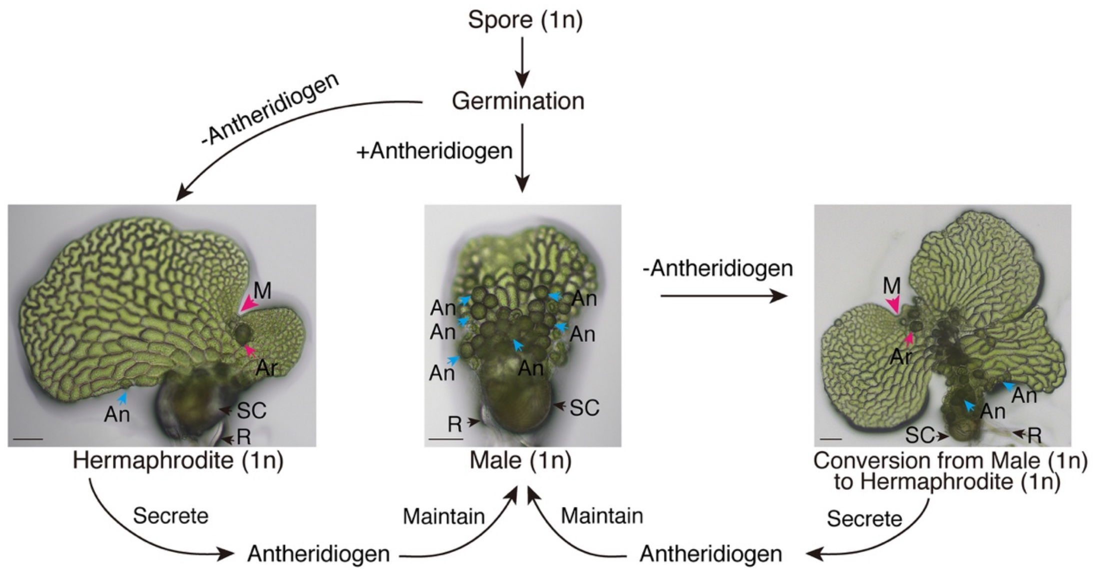

Fig. S1. Diagrams illustrating the development and morphology of Ceratopteris hermaphrodite and male gametophytes, as well as the conversion from male to hermaphrodite. After germination, the genetically identical spores (haploid, 1n) of Ceratopteris can develop into two distinct sex types. In the absence of the pheromone antheridiogen, the germinated spore develops as a hermaphrodite. The hermaphrodite (1n) contains a multicellular meristem (M, indicated by a magenta arrowhead), egg-producing archegonia (Ar, indicated by a magenta arrow), and sperm-producing antheridia (An, indicated by a blue arrow). Hermaphrodites also produce and secrete antheridiogen into the environment. In the presence of antheridiogen, the germinated spore develops into a male gametophyte. Unlike hermaphrodites, males lack a meristem and develop a large number of sperm-producing antheridia (indicated by blue arrows). Both hermaphrodites and males retain a spore coat (SC) and rhizoids (R). The maintenance of male developmental program requires continuous exposure to antheridiogen. In the absence of antheridiogen, a male can convert into a hermaphrodite, accompanying with the *de novo* formation of a meristem (M, indicated by a magenta arrowhead) and adjacent archegonia (Ar, indicated by a magenta arrow). The newly formed hermaphrodite also produces and secretes antheridiogen into the environment, ensuring that neighboring male gametophytes remain male. Scale bars: 100 µm.

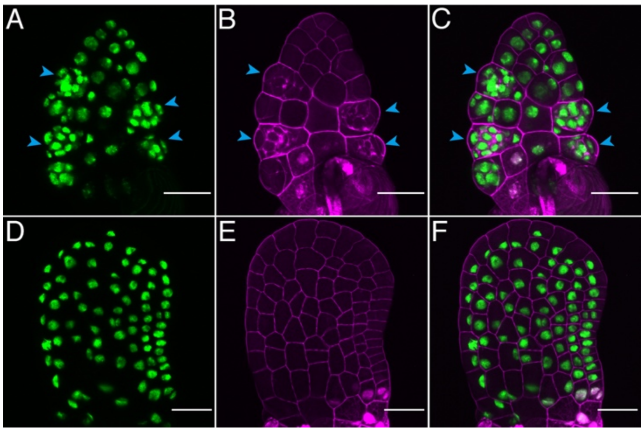

Fig. S2. Confocal imaging of Ceratopteris transgenic gametophytes expressing the *pCrUBQ10::H2B-GFP::3'CrUBQ10* reporter. Z-projection views of the representative male (3 DAG) (A-C) and hermaphrodite (3 DAG) (D-F) imaged by laser scanning confocal microscopy. Blue arrowheads (A-C) highlight antheridia in the male gametophyte. (A, D) GFP (green); (B, E) PI counterstain (magenta, showing the cell outline); (C, F) Merged channels of GFP and PI. Scale bars: 50 µm.

Development • Supplementary information

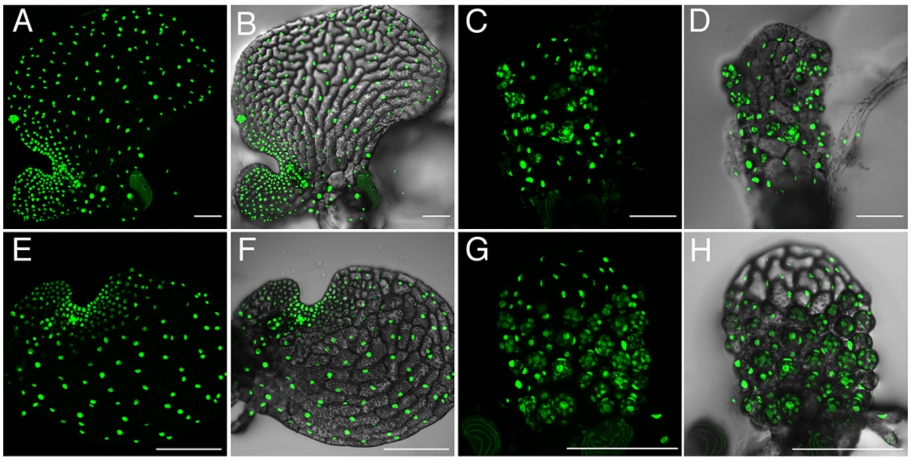

Fig. S3. Confocal imaging of Ceratopteris independent transgenic lines expressing the *pCrUBQ10::H2B-GFP::3'CrUBQ10* reporter. Z-projection views of different gametophytes (5 DAG) imaged under laser scanning confocal microscopy. (A-D) Representative hermaphroditic (A, B) and male gametophytes (C, D) from one independent transgenic line (line 14). (E-H) Representative hermaphroditic (E, F) and male gametophytes (G, H) from one independent transgenic line (line 18). (A, C, E, G): GFP channel; (B, D, F, H): Merged channels of GFP and DIC (showing the cell outline). Scale bar: 100 µm.

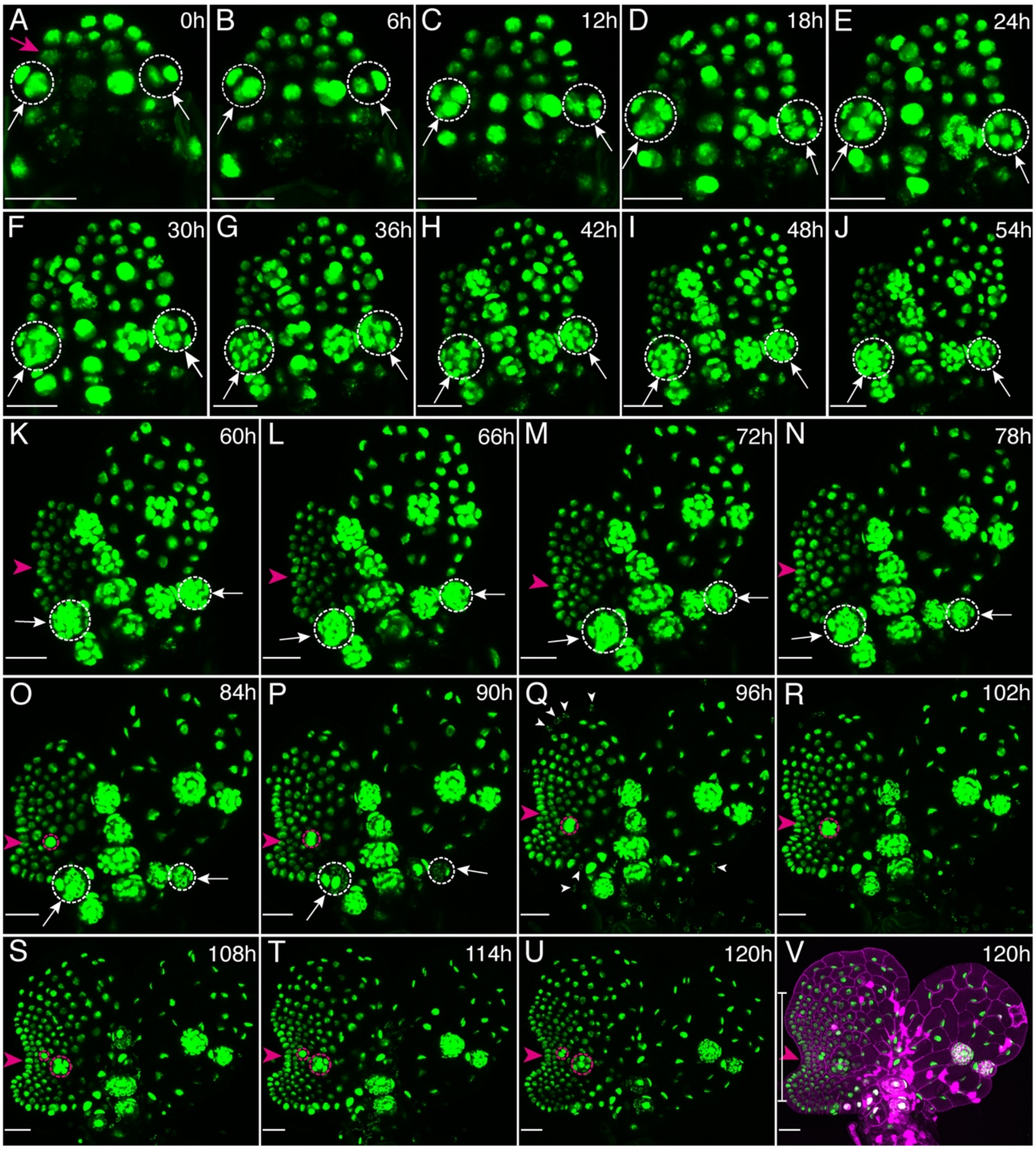

Fig. S4. Time-lapse confocal imaging of one independent sample (Sample 2) during the male-to-hermaphrodite conversion in Ceratopteris. (A-V) Z-projection views of one Ceratopteris gametophyte expressing the *pCrUBQ10::H2B-GFP::3'CrUBQ10* transgenic reporter. (A-U) At 2 DAG, the male gametophyte (Sample 2) was transferred from conditioned FM (with antheridiogen) to FM and imaged by laser confocal microscopy immediately after the transfer (0 h). The gametophyte was live-imaged every six hours up to 120 h (U) when the male had converted to a hermaphrodite with a meristem notch and the adjacent archegonia. Signals from the GFP channel are shown. White dashed circles and white arrows (A-P) highlight two representative antheridia, from initiation to maturation (releasing sperm). White arrowheads indicate motile sperm released from mature antheridia (Q). The magenta

arrow at 0 h indicates the progenitor cell contributing to the formation of a new meristem; magenta arrowheads indicate the formation of a meristem notch; magenta circles highlight the initiation and development of egg-producing archegonia. (V) The same sample at 120 h as in (U), and merged signals from the channels of both GFP (green) and PI counterstain (magenta, showing the cell outline) are shown. The white bar (V) indicates the meristem region. (A-V) Scale bars: 50 µm. At least three samples were live-imaged with the same settings and time intervals, with comparable results. Two other independent replicates are included in Fig. 2 and Fig. S5, respectively.

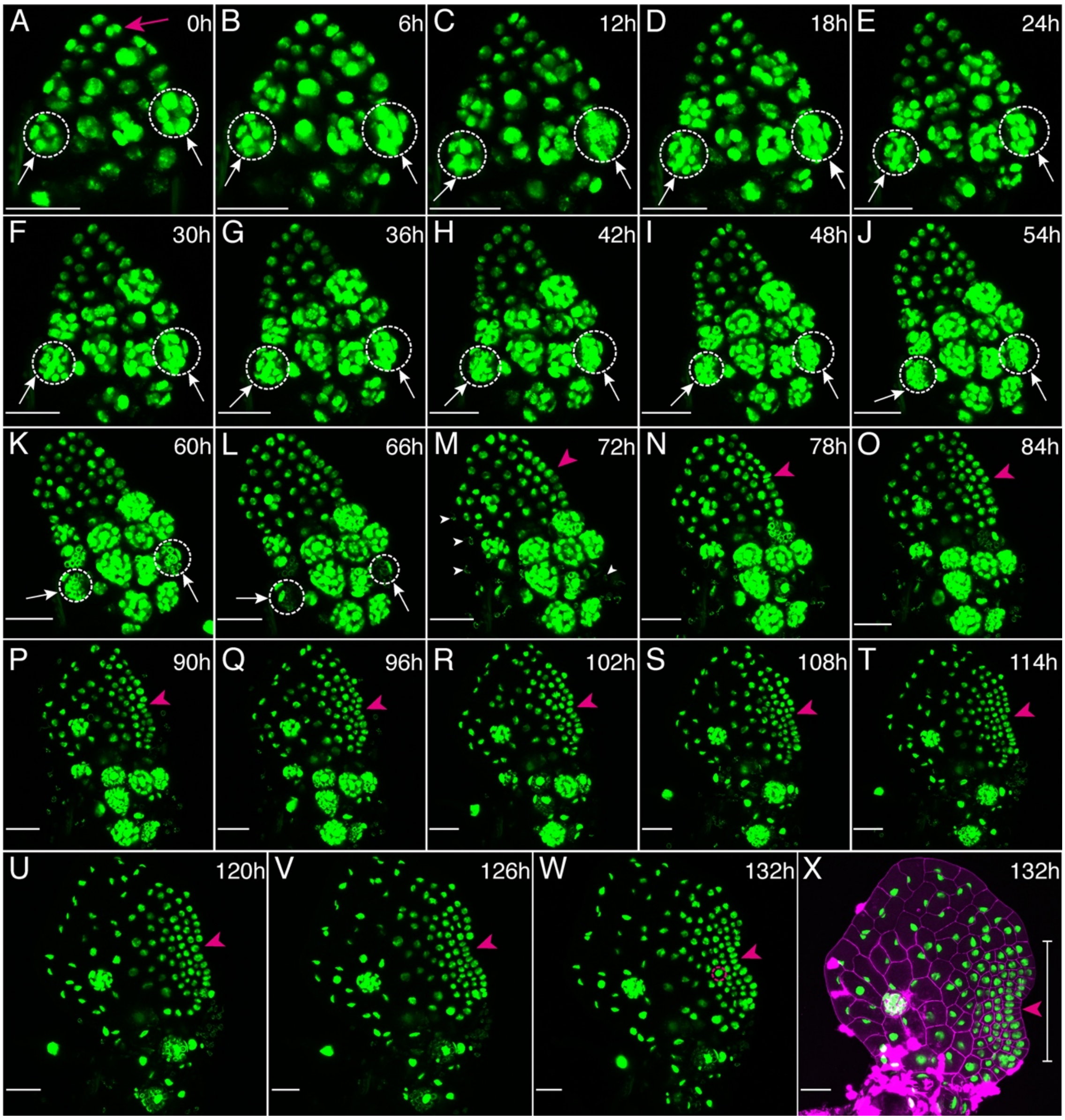

Fig. S5. Time-lapse confocal imaging of one independent sample (Sample 3) during the male-to-hermaphrodite conversion in Ceratopteris. (A-X) Z-projection views of one Ceratopteris gametophyte expressing the *pCrUBQ10::H2B-GFP::3'CrUBQ10* transgenic reporter. (A-W) At 2 DAG, the male gametophyte (Sample 3) was transferred from conditioned FM (with antheridiogen) to FM and imaged by laser confocal microscopy immediately after the transfer (0 h). The gametophyte was live-imaged every six hours up to 132 h (W) when the male had converted to a hermaphrodite with a meristem notch and the adjacent archegonia. Signals from the GFP channel are shown. White dashed circles and white arrows (A-L) highlight two representative antheridia, from initiation to maturation (releasing sperm). White arrowheads indicate the motile sperm released from mature antheridia (M). The magenta arrow at 0 h indicates the progenitor cell contributing to the formation of

Development • Supplementary information

a new meristem; magenta arrowheads indicate the formation of a meristem notch; the magenta circle highlights the initiation of an egg-producing archegonium. (X) The same sample at 132 h as in (W), and merged signals from channels of both GFP (green) and PI counterstain (magenta, showing the cell outline) are shown. The white bar (X) indicates the meristem region. (A-V) Scale bars: 50 µm. At least three samples were live-imaged with the same settings and time intervals, with comparable results. Two other independent replicates are included in Fig. 2 and Fig. S4, respectively.

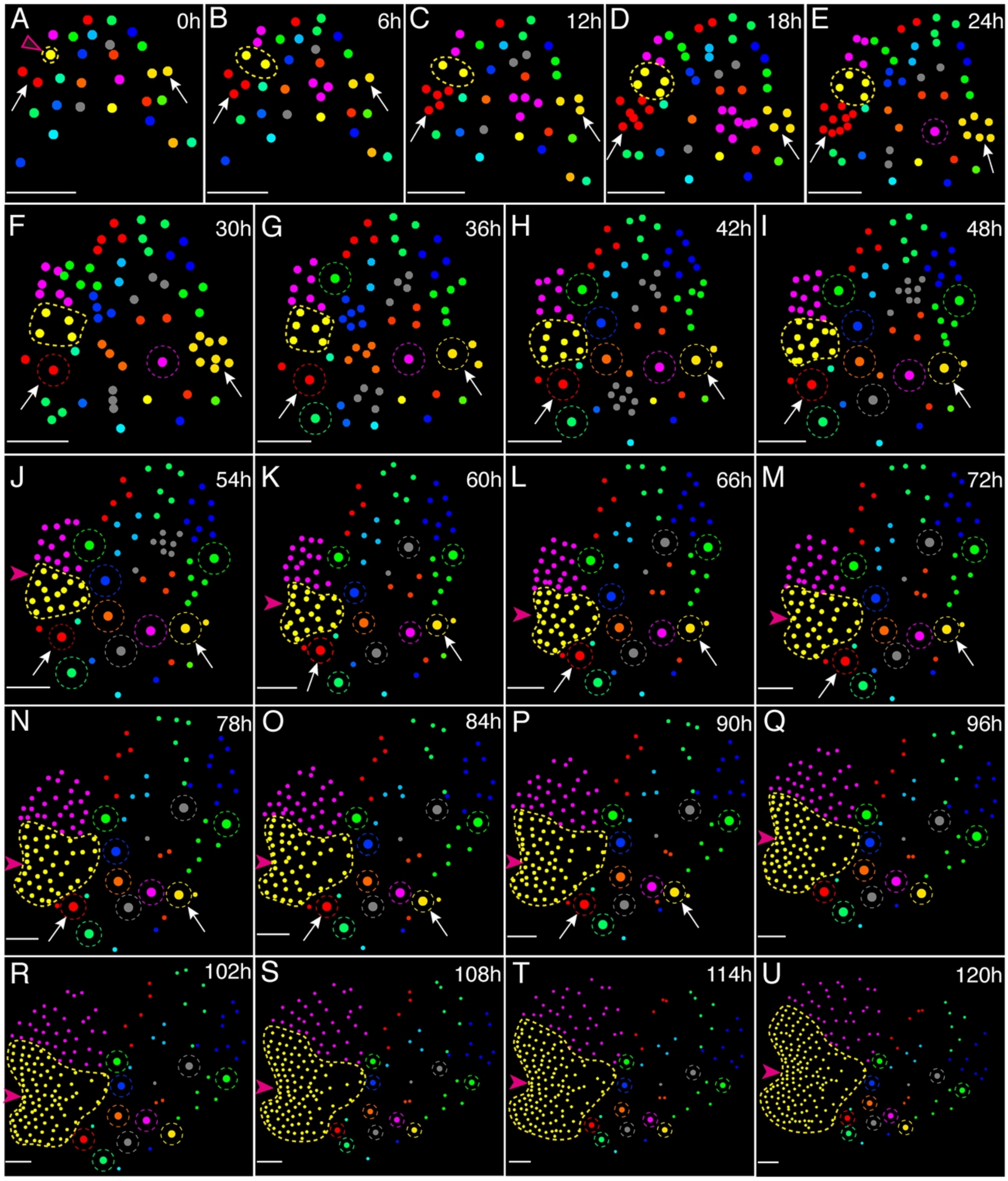

Fig. S6. Cell lineage dynamics of *de novo* meristem formation during the male-to-hermaphrodite conversion in Sample 2. (A-U) Nuclei in the confocal images of Fig. S3A-U (Sample 2) were segmented and labeled with unique IDs. Each dot represents the location of an individual nucleus in the confocal images. Each dashed circle in (E-U) represents the location of an individual mature antheridium, which forms a multiple-nuclei 3D structure. Individual nuclei at 0 h are labeled with different colors as a reference for the lineage analysis, and the same color is assigned to progeny cells derived from the same cell in images taken at 6-120 h. The magenta open arrowhead at 0 h (A) indicates the meristem progenitor cell (MPC, colored in yellow) of the lineage contributing to the newly

Development • Supplementary information

formed meristem. Yellow dashed circles (A-U) indicate the MPC lineage. Magenta arrowheads (J-U) indicate *de novo* formation of a multicellular meristem. White arrows (A-P) indicate the two representative antheridia also shown in Fig. S3A-P. Scale bars: 50 µm. Three independent samples were analyzed, showing comparable results. Results of lineage dynamics for the other two samples are included in Fig. 3 and Fig. S7, respectively.

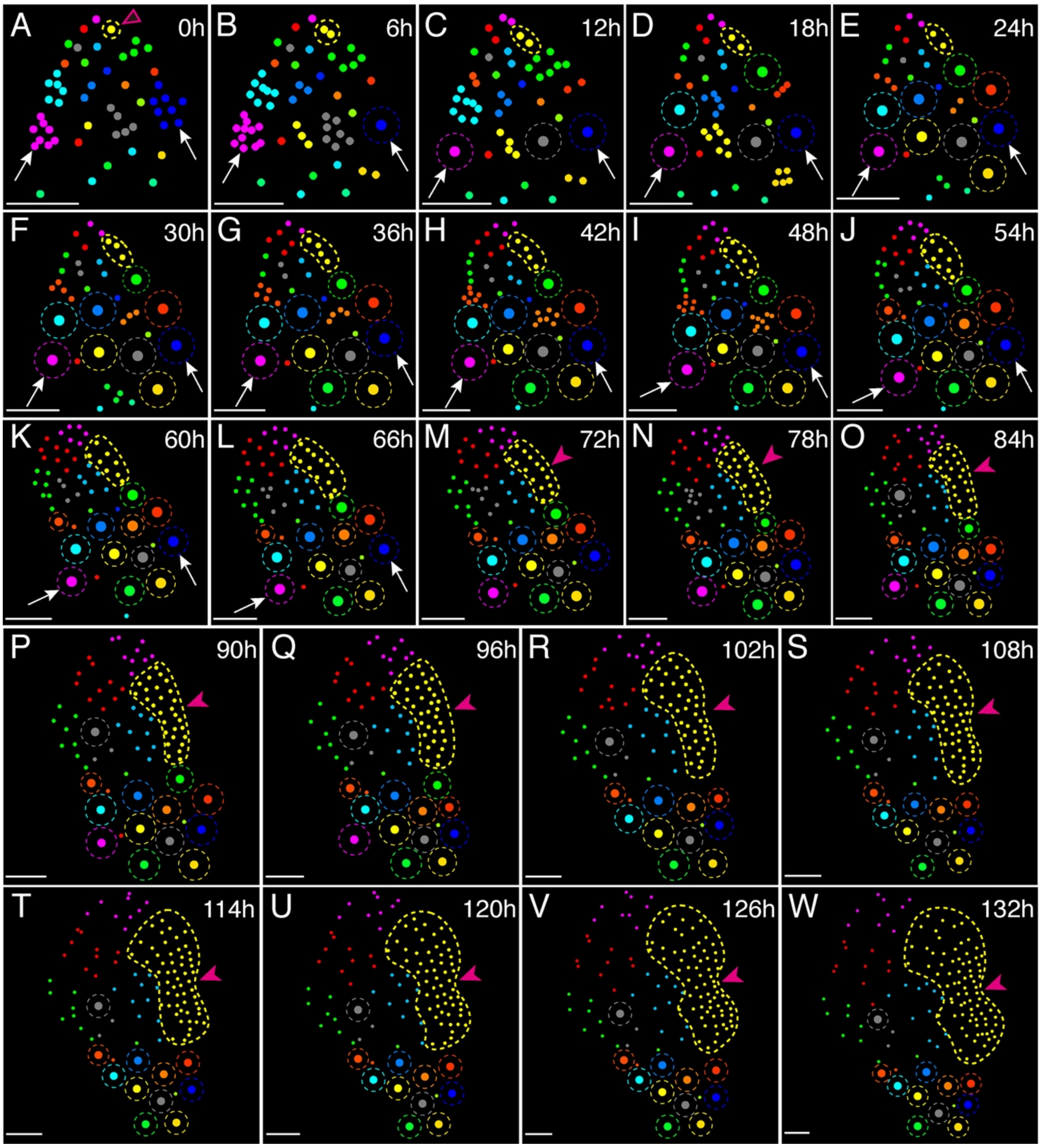

Fig. S7. Cell lineage dynamics during the male-to-hermaphrodite conversion in Sample 3. (A-U) Nuclei in the confocal images of Fig. S4A-W (Sample 3) were segmented and labeled with unique IDs. Each dot represents the location of an individual nucleus in the confocal images. Each dashed circle in (B-W) represents the location of an individual mature antheridium, which forms a multiple-nuclei 3D structure. Individual nuclei at 0 h are labeled with different colors as a reference for the lineage analysis, and the same color is assigned to progeny cells derived from the same cell in images taken at 6-132 h. The magenta open arrowhead at 0 h (A) indicates the meristem progenitor cell (MPC, colored in yellow) of the lineage contributing to the newly formed meristem. Yellow dashed circles (A-W) indicate the MPC lineage. Magenta arrowheads (M-W) indicate *de novo* formation of a multicellular meristem. White arrows (A-L) indicate the two representative antheridia also shown in Fig. S4A-L. Scale bars: 50 µm. Three independent samples were analyzed, showing comparable results. Results of lineage dynamics for the other two samples are included in Fig. 3 and Fig. S6, respectively.

Development • Supplementary information

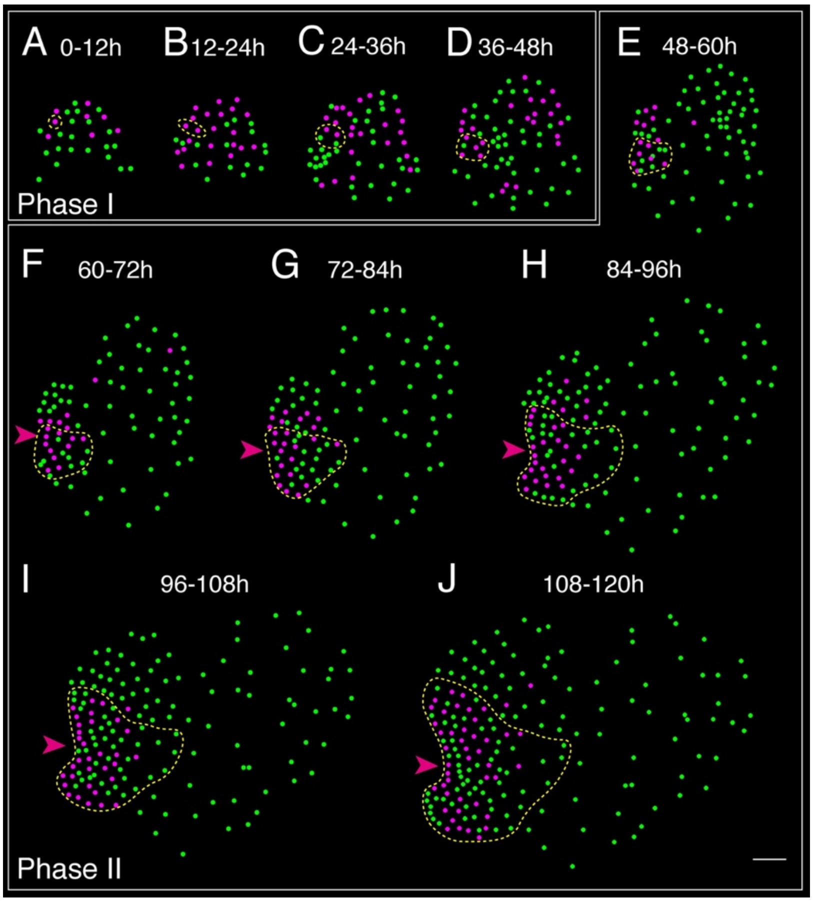

Fig. S8. Cell division dynamics during the male-to-hermaphrodite conversion in Sample 2. Each colored dot indicates the nucleus of confocal images (Sample 2), except for mature antheridia, which were not included division quantification. (A-J) magenta dots indicate cells that underwent division, and green dots indicate cells that remained undivided during the indicated 12-h period. The magenta arrowheads (F-J) indicate the initiation and development of a multicellular meristem. Yellow dashed circles indicate the MPC lineage. Scale bar: 50 µm. Three independent samples were analyzed, showing comparable results. Results of cell division dynamics for the other two samples are included in Fig. 4 and Fig. S9, respectively. Phase I and Phase II indicate the two main developmental stages during the male-to-hermaphrodite conversion.

Development • Supplementary information

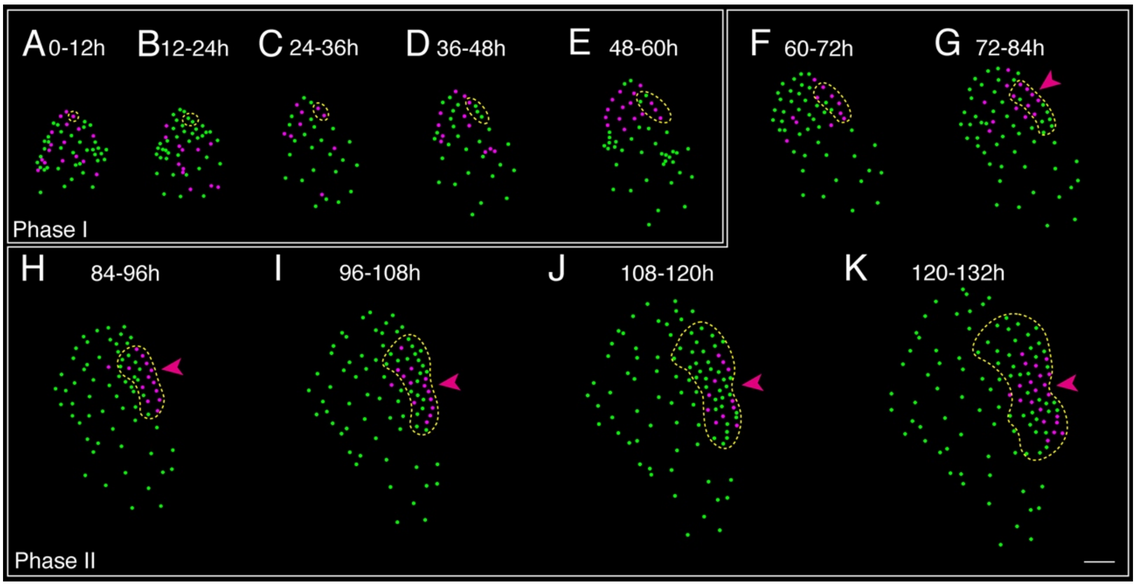

Fig. S9. Cell division dynamics during the male-to-hermaphrodite conversion in Sample 3. Each colored dot indicates the nucleus of confocal images (Sample 3), except for mature antheridia, which were not included in division quantification. (A-K) magenta dots indicate cells that underwent division, and green dots indicate cells that remained undivided during the indicated 12-h period. The magenta arrowheads (G-K) indicate the initiation and development of a multicellular meristem. Yellow dashed circles indicate the MPC lineage. Scale bar: 50 µm. Three independent samples were analyzed, showing comparable results. Results of cell division dynamics for the other two samples are included in Fig. 4 and Fig. S8, respectively. Phase I and Phase II indicate the two main developmental stages during the male-to-hermaphrodite conversion.

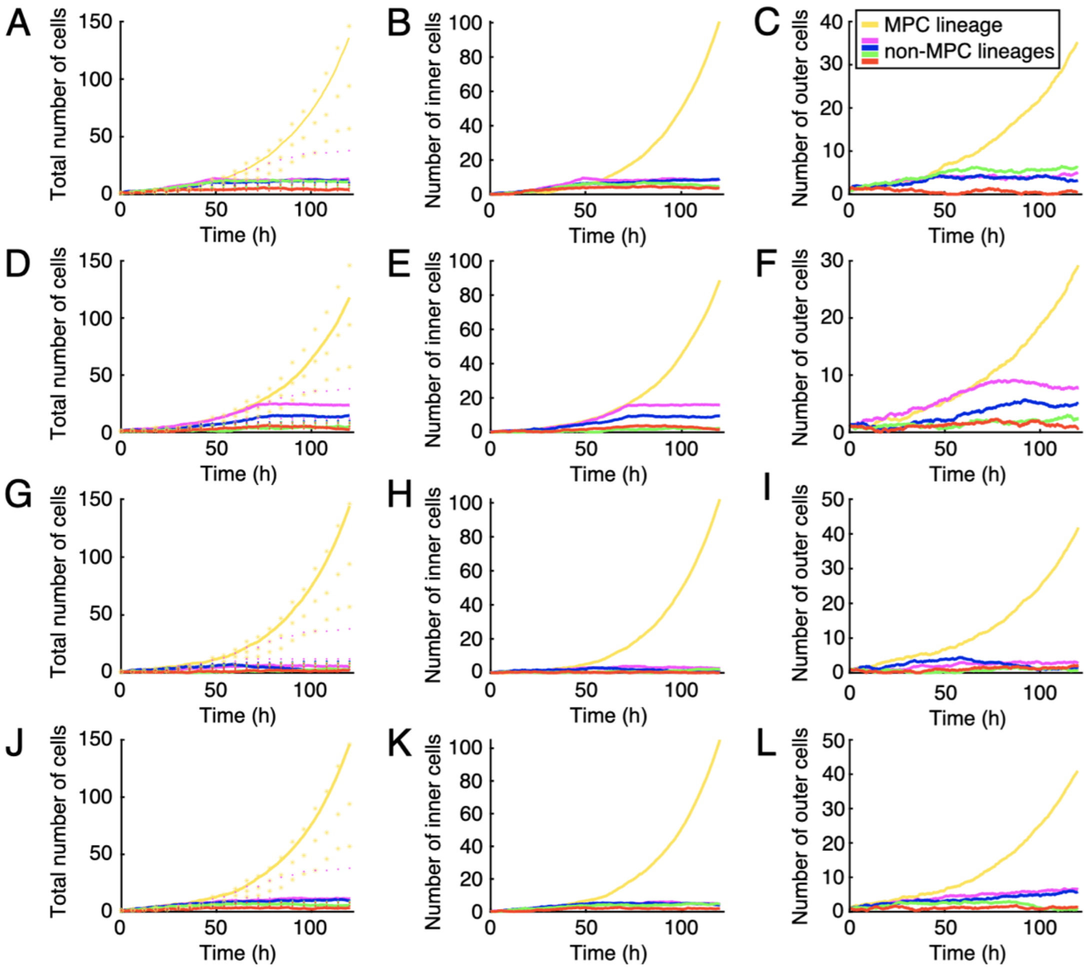

Fig. S10 Example model (threshold version) simulations. The main text included average cell numbers generated by a simple mathematical model, based on 1000 simulations for each condition (treated and untreated). For more intuition, four example untreated simulations of the threshold version of the model are presented here, with each row corresponding to one example. (A-C) Example 1, (D-F) example 2, (G-I) example 3, and (J-L) example 4 illustrate representative variability in the model simulations. In all cases shown (A, D, G, J), one lineage clearly emerged as dominant. This is also visible in the number of inner cells (B, E, H, K) and outer cells (C, F, I, L) for these examples. The points in (A, D, G, J) refer to empirical measurements (Fig. 5D-F); specifically, for each of the three samples, the cell numbers for its largest lineage (by number of cells at the final time, 120 h) are plotted in yellow, its second largest lineage in magenta, its third in blue, its fourth in green, and its fifth in red.

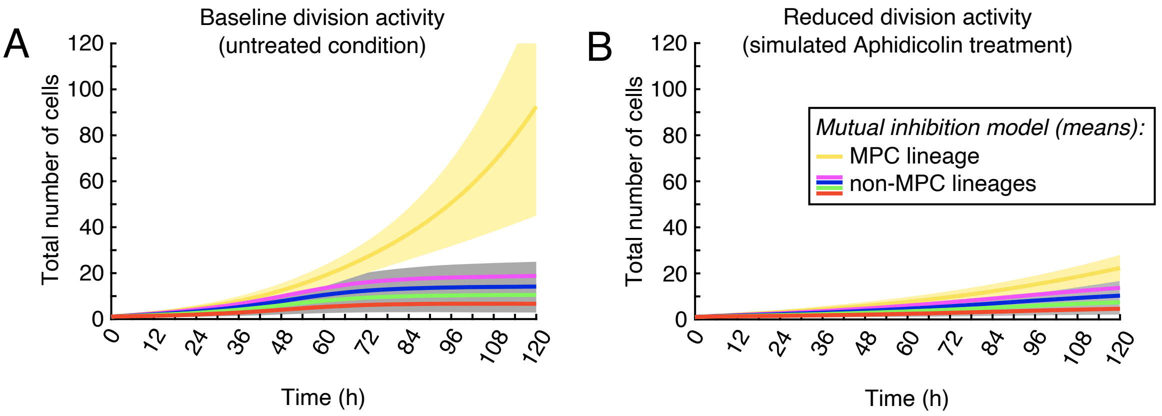

Fig. S11. Mathematical modeling of lineage dynamics without threshold behavior. The main text included average cell numbers generated by a simple mathematical model that prescribes inhibitory signals once a threshold difference in division activity between lineages is reached. An alternative version of the model (the "mutual inhibition version") specifies that all lineages inhibit the other lineages at all times, but inhibition strength depends on division activity. (A) The mutual inhibition version of the model was simulated 1000 times and the mean cell numbers were reported in time. Computing the cell-number curves was done in the same way as described in the caption of Fig. 8. (B) To simulate Aphidicolin treatment, the mutual inhibition version of the model was simulated 1000 times with the division rate r reduced by half for all of the lineages. The yellow shaded region indicates plus or minus one standard deviation for the largest lineage; the grey shaded area indicates the approximate region occupied by the plus or minus one standard deviation curves for all of the other lineages.

Development • Supplementary information

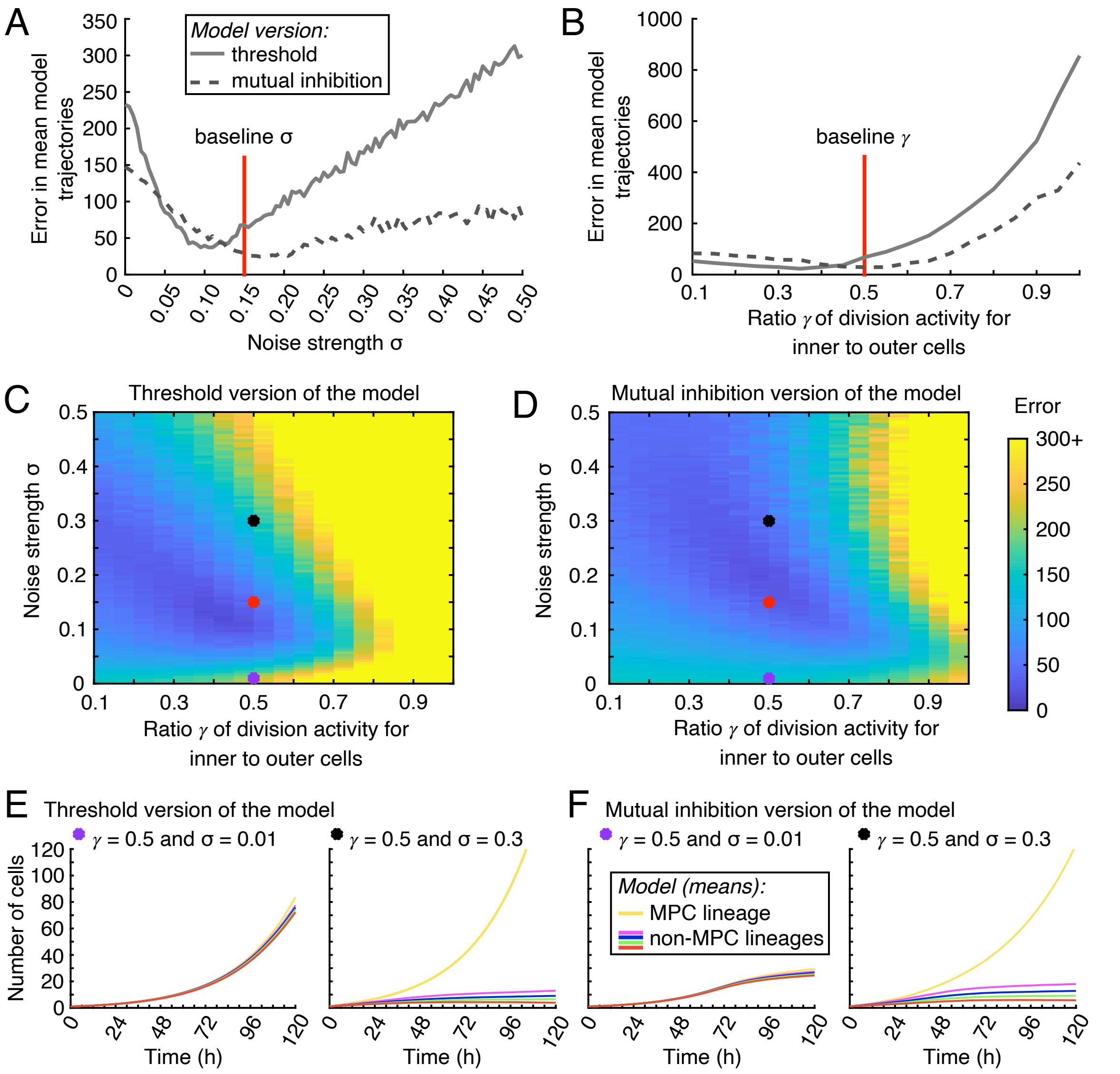

Fig. S12. Effects of parameters on model results. (A) The baseline noise strength in all model simulations was $\sigma = 0.15$, and the solid and dashed curves show how the difference between mean in silico and in vivo cell-number trajectories varies with noise strength. (B) The parameter γ prescribes the ratio of the division rate for inner to outer cells, with a baseline value of $\gamma = 0.5$ motivated by Fig. 7G. Both versions of the simple mathematical model are insensitive to this parameter as long as it is sufficiently low, according to the mean cell-trajectory curves. (C-D) Heatmaps show the error between model and data as a function of different (γ, σ) pairs, for the threshold version of the model and mutual inhibition version of the model, respectively. Red circle indicates baseline parameter values. (E-F) Simulations with low and high noise strength, for the threshold and mutual inhibition versions of the model, respectively, show that low noise strength prevents establishment of a MPC lineage in the timeframe considered. On the other hand, higher noise strength of $\sigma = 0.3$ leads to mean cell numbers

Development • Supplementary information

in the leading lineage that are too high. The results (A-D) are based on 300 model simulations under each parameter set, and mean cell numbers are reported. The results (E-F) are based on 1000 model simulations under each parameter set, with mean cell numbers shown. Error in (A-D) refers to the square root of the sum of squared differences (across 5 lineages and 21 measurement times) between the mean number of cells in silico and the mean number of cells *in vivo*.

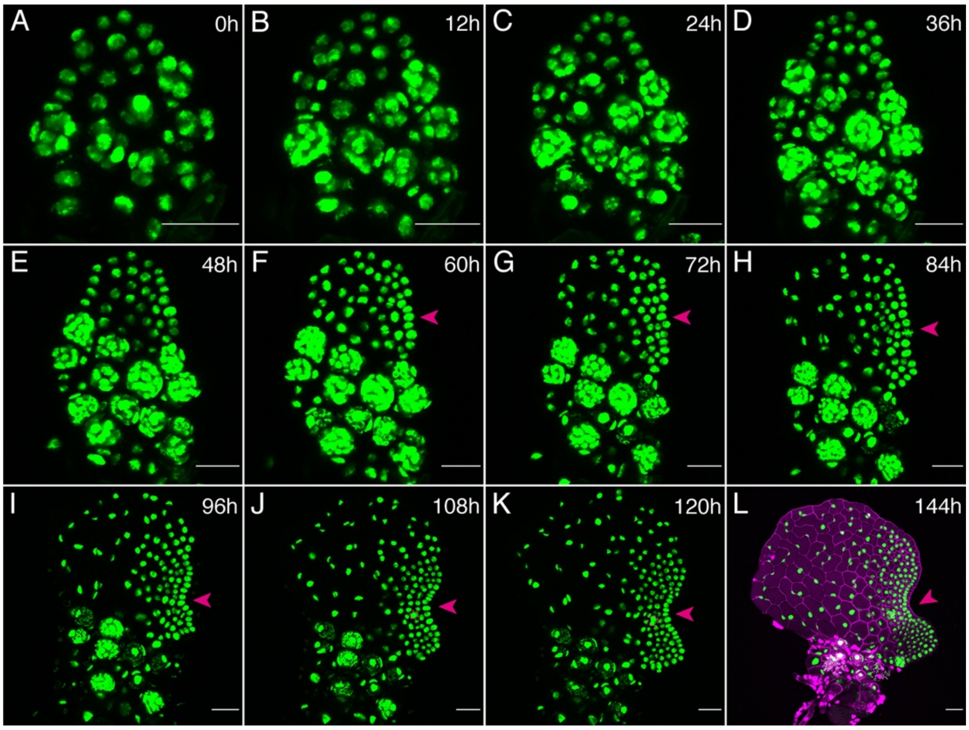

Fig. S13. Time-lapse confocal imaging of male-to-hermaphrodite conversion in the mock control. (A-L) Z-projection views of GFP signals (green) taken from one representative male gametophyte expressing the *pCrUBQ10::H2B-GFP::3'CrUBQ10* transgenic reporter. (A) At 2 DAG, the male was transferred from CFM to FM supplemented with 0 µg/mL Aphidicolin (the mock control) and immediately imaged by laser confocal microscopy (0 h). (B-L) The gametophyte was live-imaged every 12 hours up to 144 h when the male had converted to a hermaphrodite with a fully established meristem and archegonium. Magenta arrowheads (F-L) indicate the newly formed meristem. (L) Merged GFP (green) and PI (magenta) channels. (A-L) Scale bar: 50 µm. At least three samples were live-imaged with the same settings and time intervals, showing comparable results.

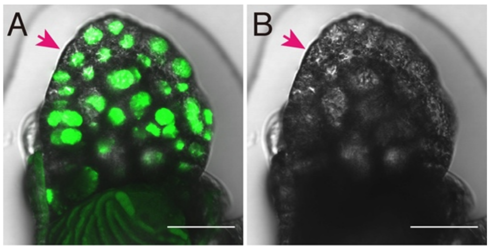

Fig. S14. Confocal images of Sample 1 at 0 h. (A) Merged channels of GFP (green) and DIC (gray, showing cell outlines). (B) DIC channel. Red arrows indicate the MPC at 0 h. The Z-projection view of GFP signals for Sample 1 is presented in Fig. 2A. Scale bars: 50 µm.

Development • Supplementary information

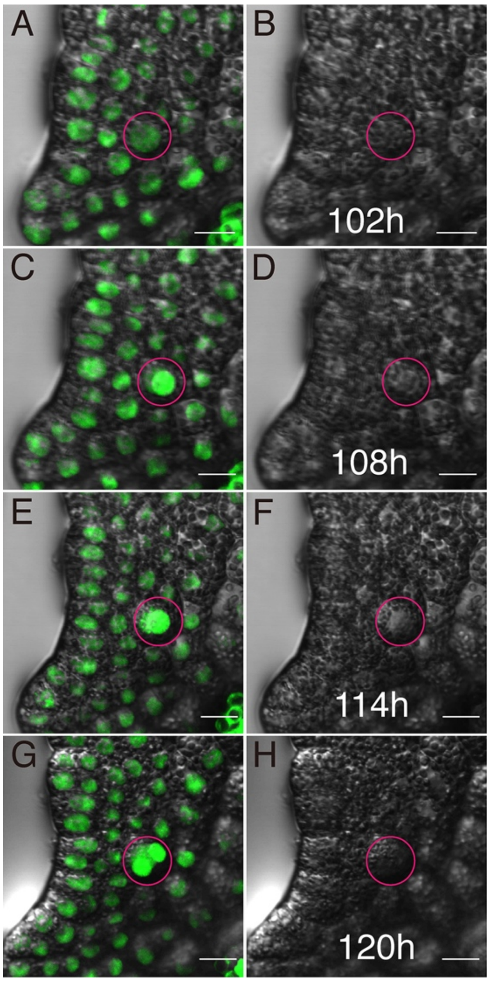

Fig. S15. Archegonium development in the newly formed hermaphrodite (Sample 1). (A-H) Zoomed-in images of Sample 1 from 102 to 120 h (as shown in Fig. 2R-U). (A, C, E, G) Merged channels of GFP (green) and DIC (gray, showing cell outlines). (B, D, F, H) DIC channel. Magenta circles (A-H) indicate the developing archegonium captured at different time points. Scale bars: 20 µm.

Development • Supplementary information

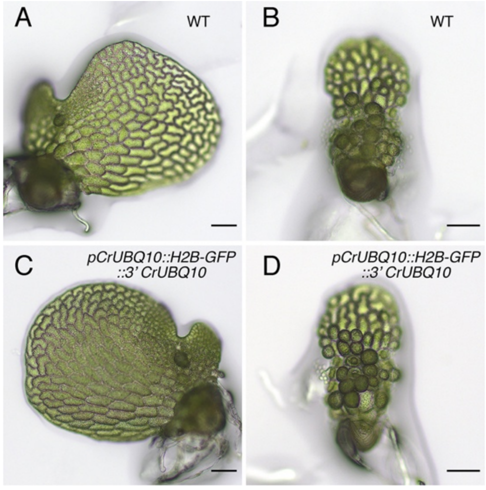

Fig. S16. Gametophytes of the wild-type and transgenic line at 5 DAG. (A, B) Light micrographs of a hermaphrodite and a male gametophyte from the Ceratopteris wild-type (Hn-n) at 5 DAG. (C, D) Light micrographs of a hermaphrodite and a male gametophyte from the Ceratopteris *pCrUBQ10::H2B-GFP::3'CrUBQ10* transgenic line (line 10) at 5 DAG. Scale bars: 100 µm.

Table S1. Total cell number in three samples

Available for download at
https://journals.biologists.com/dev/article-lookup/doi/10.1242/dev.204411#supplementary-data

Table S2. Cell division events in three samples

Available for download at
https://journals.biologists.com/dev/article-lookup/doi/10.1242/dev.204411#supplementary-data

Table S3. Cell division events in the outermost and second layers of three samples

Available for download at
https://journals.biologists.com/dev/article-lookup/doi/10.1242/dev.204411#supplementary-data

Development • Supplementary information

Table S4. Anticlinal and periclinal division events in the outermost layer of newly formed meristems across three samples

Available for download at
https://journals.biologists.com/dev/article-lookup/doi/10.1242/dev.204411#supplementary-data

Table S5. Total cell number in four samples treated with Aphidicolin

Available for download at
https://journals.biologists.com/dev/article-lookup/doi/10.1242/dev.204411#supplementary-data

Development • Supplementary information

Table S6. Summary of the parameters in the mathematical model and the parameters involved in its numerial implementation. In this simplified model, the parameteres α, K, ε^{in}, and ε^{out} are described as having units of number of cells, but note that these parameters may take on non-integer values. Alternatively, they could be thought of as quantifying the concentration of a diffusing signal or morphogen produced by the cells.

Parameter	Value	Description
α	1 cell	Threshold value associated with declaring one lineage the current MPC canddiate
K	4 cells (threshold version) or 8 cells (mutual inhibition version)	Concentration parameter in the Hill-type function term for repressing division
m	4 (unitless) (threshold version) or 8 (unitless) (mutual inhibition version)	Coefficient in the Hill-type function term for repressing division
r	$0.05\ \text{h}^{-1}$ for baseline $0.025\ \text{h}^{-1}$ for treatment	Division rate of outer cells
γ	0.5 (unitless)	Reduction in the division rate of inner cells (inner cells divide at a rate of γr according to the model)
σ	0.15 (unitless)	Noise strength in the stochastic differential equations
ε^{in}	0 cells	Lower bound for the minimum number of inner cells to enforce positivity
ε^{out}	0.001 cells	Lower bound for the minimum number of outer cells to enforce positivity
Δt	0.1 h	Time step in the numerical implementation

Note to Table S6 on numerical implementation of the mathematical model

The stochastic differential equations were solved in MATLAB using the Euler–Maruyama method. In all cases, the initial condition consisted of five lineage populations, each with zero inner cells and one outer (marginal) cell at time $t = 0$ hours. Alternatively, in this simple model, the population variables can be thought of as capturing the concentration of a diffusing signal or morphogen produced by the cells (i.e., concentrations that are proportional to cell number). The time step was $\Delta t = 0.1$ h, and the noise strength was $\sigma = 0.15$ in all of the model simulations except for the parameter tests in Fig. S12. To enforce positivity, after each time step, if the number of outer cells of lineage i, namely $P_i^{\text{out}}(t)$, was less than $\varepsilon = 0.001$, then the value of $P_i^{\text{out}}(t)$ was set to ε. Similarly, at each time step, if the number of inner cells of lineage i was less than 0, then the value of $P_i^{\text{in}}(t)$ was set to 0. A positive, though very small, threshold for $P_i^{\text{out}}(t)$ was used to prevent any stochastic simulations from resulting in very few

Development • Supplementary information

cells. The errors between model and data shown in Fig. S12 were computed according to the following equation:

$$\text{error} = \sqrt{\sum_i^5 \sum_{\tau=1}^{21} \left(\tilde{P}_i(t_\tau) - \tilde{C}_i(t_\tau)\right)^2},$$

where the first sum is over the five cell lineages, the second sum is over the 21 measurement times $\{t_\tau\}$ at 6 h intervals from 0 h to 120 h, $\tilde{P}_i(\cdot)$ denotes the mean total number of cells (inner and outer) in the ith lineage across the model simulations, and $\tilde{C}_\mathrm{i}(\cdot)$ denotes the mean total number of cells in the ith lineage across the experimental replicates. The model and implementation parameters are in Supplementary Table S6, and the model code is publicly available in a GitLab repository (https://gitlab.com/alexandriavolkening/population-model-for-meristem-dynamics-in-ferns).

Development • Supplementary information
