# Supplementary material for: Single cell-derived multicellular meristem: insights into male-to-hermaphrodite conversion and de novo meristem formation in Ceratopteris
Source: Development. 2025 Feb 13;152(3):DEV204411. doi: 10.1242/dev.204411 (PMC11883269; doi:10.1242/dev.204411)
Supplement: Supplementary file 1 [file develop-152-204411_review_history.pdf]

Single cell-derived multicellular meristem: insights into male-to-hermaphrodite conversion and *de novo* meristem formation in *Ceratopteris*

Xi Yang, An Yan, Xing Liu, Alexandria Volkening and Yun Zhou
DOI: 10.1242/dev.204411

Editor: Dominique Bergmann

Review timeline
Original submission: 22 September 2024
Editorial decision: 5 November 2024
First revision received: 18 December 2024
Accepted: 4 January 2024

Original submission

First decision letter

MS ID#: dev.204411

MS TITLE: Single Cell Derived Multicellular Meristem: Insights into Male-to-Hermaphrodite Conversion and De Novo Meristem Formation in Ceratopteris

AUTHORS: Xi Yang, An Yan, Xing Liu, Alexandria Volkening and Yun Zhou

Dear Dr Zhou,

I have now received all the referees reports on the above manuscript, and have reached a decision. The referees' comments are appended below, or you can access them online: please go to .

The overall evaluation is positive and we would like to publish a revised manuscript in Development, provided that the referees' comments can be satisfactorily addressed. You will see that Reviewer 1 and 2 suggest a number of changes to the text and presentation of data, but Reviewer 3 also suggests additional modeling and/or experimentation. It is important to use the modeling effectively to complement the experiments, so please pay particular attention to these suggestions. Please attend to all of the reviewers' comments in your revised manuscript and detail them in your point-by-point response. If you do not agree with any of their criticisms or suggestions explain clearly why this is so. If it would be helpful, you are welcome to contact us to discuss your revision in greater detail. Please send us a point-by-point response indicating your plans for addressing the referees' comments, and we will look over this and provide further guidance.

Reviewer 1

Advance summary and potential significance to field

The de novo formation of a meristem has been well described during the embryogenesis of flowering plants such as Arabidopsis thaliana. However, this is technically difficult due to the enclosure of the embryo within the seed coat, and the meristem structure and regulation of the diploid embryo is not representative of other major land plant lineages.

© 2025. Published by The Company of Biologists under the terms of the Creative Commons Attribution License (https://creativecommons.org/licenses/by/4.0/).

In this paper, the authors describe the de novo formation of meristems in the haploid stage of the fern, Ceratopteris richardii. The free-living fern gametophyte can convert from a meristem-less male to a hermaphrodite with an indeterminate marginal meristem. Whilst the cell division patterns of the hermaphrodite marginal meristem have already been described (Geng et al., 2022), this paper adds a new description of how meristem formation occurs during the male-female transition. They find that a new meristem is initiated from a single cell, which contrasts to previous descriptions in hermaphrodite gametophytes in which multiple cells contribute to the meristem (Geng et al., 2022). For me, the most interesting and novel finding is that this dominant cell lineage can arise from stochastic variations in cell division across the tissue, provided that the dividing marginal cells produce a mobile inhibitory signal. This mathematical explanation of stem cell niche formation provides a foundation to test novel mechanisms of tissue patterning.
I would like to commend the authors for the well written text, laborious 6hr interval timelapse experiments, and inclusion of technical repeat data.

Comments for the author

1) Many of the techniques and conclusions are similar to the results of Geng et al., 2022. For example, both cases use the term "Meristem progenitor cell (MPC)", and describe different "Phases" of meristem growth (Phase I, II, III for Geng et al., and Phase I and II for Yang et al). It would be helpful to include a comparison of these terms and findings throughout the text, to understand how these two processes- meristem formation in the hermaphrodite gametophyte and meristem formation during the male-hermaphrodite transition- are similar and different, and emphasise which findings are novel.

2) The introduction focuses primarily on the sex determination of the gametophyte. Since the paper describes the development and structure of the meristem, it would be useful to include a short paragraph about meristem structure (e.g. apical meristem, marginal/lateral meristem, single-celled vs multicellular meristem) in different ferns, and what has already been shown. E.g. Geng et al., 2022, Wu et al., 2022a, Wu et al., 2022b, Bartz and Gola, 2018, Takahashi et al., 2009. Some of these papers were referenced, but the description is very vague (line 65-66).

3) Throughout the text, the authors describe the process of meristem formation. However, it is unclear to me what defines the "meristem". During its formation, is there a landmark feature (e.g. notch shape? Frequency of division?) that marks the transition from a forming meristem to a formed meristem? In the figures, meristems are marked with magenta arrowheads, however it is difficult to understand when the authors are able to classify the structure as a meristem. Perhaps a clear definition of the marginal meristem in the introduction would help clarify this.

4) Does the gametophyte prothallus only consist of one cell layer? This is implied by the type of analysis that is performed, but isn't explicitly stated. This could be added to the paragraph suggested in comment 2.

5) Line 119-121: Why were new reporter lines generated? What advantage do these lines have over previously described reporter lines? Since these lines are new, it would be good to include a description and photos of how these lines grow in comparison to wild type. Do they have any developmental defects?

6) Line 138-line 140: Live samples were imaged every 6 hours. Did imaging affect the rate of growth of the gametophytes?

7) Line 153-156: Are any more antheridia initiated after the transfer to antheridiogen free media? Or is it just that pre-existing antheridia are maturing?

8) Line 268-274: For the computational modelling, four assumptions are made. Whilst the assumptions seem logical, they are not all experimentally validated. Is it true that anticlinal and periclinal divisions are equally likely? Could this be quantified from your existing cell division data? Could the authors show the results of the model if each of the assumptions was excluded?

© 2025. Published by The Company of Biologists under the terms of the Creative Commons Attribution License (https://creativecommons.org/licenses/by/4.0/).

9) Line 362-366: The authors state that non-meristematic cells become meristematic during de novo induction of meristems. Is there a marker of differentiation that could be tracked after transfer to antheridiogen free media, to confirm if this is occurring in the gametophyte? Or perhaps a stain for a component that may be present in differentiated cells but not meristematic cells?

10) Woudenberg et al., 2024 (Development, https://doi.org/10.1242/dev.203026) recently showed that auxin inhibits cell division in the fern gametophyte. Perhaps this paper should be cited in the discussion as a potential mobile inhibitory signal derived from the forming marginal meristem?

Minor points:

11) Line 385: Give some examples of the signals from the epidermis in flowering plants.

12) Methods: If possible, include the light spectra of the growth cabinets to improve experimental reproducibility. At the very least, record the total light intensity.

13) Line 466: "29C"-> "29°C"

14) Figure 1: The frequency of conversion from male-hermaphrodite could be included here, to show how reproducible the induction is.

15) Figure 2: Why are only two antheridia highlighted? Can't the same antheridia be marked as in Figure 3?

16) Figure 3: The grey antheridium in Panel O disappears from panel P onwards. The rest are present throughout. Is there a reason for this?

17) Figure 6: Label Sample 1, 2 and 3 as in other figures.

18) Figure 7: The figure legend is very repetitive. Is there a more concise way to organise it?

19) Figure 9: D-F in the figure show conversion to hermaphrodites, but the legend says they are remaining as males (line 751). Swap either the order of the panels or the text.

20) Figure 10: The dashed circles are very thin and difficult to see. Increase the line thickness.

<u>Reviewer 2</u>

Advance summary and potential significance to field

This manuscript by Yang et al. reports the identification of the cellular mechanisms by which the ameristic antheridium (the male organ) regenerates a meristem and eventually forms a hermaphrodite in Ceratopteris using confocal-based long-term, non-invasive time-lapse imaging and computational image analysis. Despite many outstanding questions in plant sexual reproduction, this study presents the fern Ceratopteris as an excellent system for addressing fate changes between male and female gametophytes. The findings reveal that in the absence of antheridiogen, a single non-antheridium cell acts as the meristem progenitor cell to reform a multicellular meristem through active cell division in the marginal layer, thus converting the ameristic antheridium into the meristic hermaphrodite, which are capable of producing both male and female gametophytes. Simulations from a proposed mathematical model suggest cells derived from proliferation of one lineage of cells inhibit the division of cells from other lineages once a certain threshold is reached, attributed to stochastic variation in cell division timing. Furthermore, the authors show that DNA synthesis is necessary for the establishment of the meristem progenitor cell (MPC) and the antheridium-to-hermaphrodite conversion. These results not only shed light on the cellular mechanism underlying conversion of sex types in ferns, but also address long-standing questions in plant developmental biology using novel tools, making this study significant. The presented data are voluminous, and the conclusions are sound. However, this reviewer lacks

© 2025. Published by The Company of Biologists under the terms of the Creative Commons Attribution License (https://creativecommons.org/licenses/by/4.0/).

expertise in imaging techniques and therefore has no comments on the experimental design or execution in that area.

Comments for the author

Some suggestions and Clarifications:

1. Antheridiogen promotes the formation of antheridia (male organs), which lack meristem. In the absence of antheridiogen, antheridia gain the ability to regenerate the meristem, leading to expansion of prothallus and the production of both egg-bearing archegonia (female organs) and antheridia. This reviewer is not clear whether hermaphrodites produce antheridia before archegonia or simultaneously. The conversion of the male organ into a hermaphrodite appears to resemble the process of an arrested hermaphrodite resuming its development to complete its growth. Whether or not this is the case, it would be helpful for readers to better understand the discoveries if the authors could compare these two developmental processes.

2. Fig. 1 shows that hermaphrodites exhibit a heart-shaped prothallus at 15 DAG (Fig. 1D). When a male gametophyte (2 DAG) was transferred to an antheridiogen-free growth medium, it seems taking a longer time to form a heart-shaped prothallus, as suggested by the difference in prothallus size when comparing images in Figs. B and C to those in Figs. J and K. This should be clarified.

3. In Fig. 2., please define how the egg-producing archegonia were defined.

4. This reviewer may have missed key details in the manuscript, so it would be helpful if the authors elaborate on how the MPC and cell lineages were defined in Figs. 3 and 4.

5. A couple of questions that are not required to secure the publication of this manuscript but might be of interest: (1) Is the remaining portion of the male organ, without antheridia, still capable of converting into a hermaphrodite? (2) If the meristem is impaired, can hermaphrodites convert into antheridia?

Reviewer 3: In this manuscript, the authors examined the de novo induction of new meristems in Ceratopteris during the conversion from males to hermaphrodites. A combination of experiments and mathematical modeling are adopted. An interesting finding is that a single non-antheridium cell from the male body serves as the MPC in the new formed hermaphrodite, and the MPC lineage exclusively contributes to the entire formation of the new meristem. Correspondingly, a math model is developed to test this phenomenon and so to explore the mechanism behind. Overall, I think the results presented in this manuscript is interesting, and can offer great insights not only in plant biology, but also in development. However, I do have some concerns. I'm not an expert in Ceratopteris biology so I will leave the biology part for other people to comment, below I'll mainly comment based on the modeling part.

1. I have concerns on how the modeling results support the experimental findings. As is pointed out by the authors themselves, the model used here is a simple one - which is fine with me, as long as the model can help elucidate the underlying mechanisms of the system. I feel like the model here is designed with the only goal that it behaves the same as what is observed from experiments, but very little efforts are made to touch the mechanisms behind. To be specific, the logic of the research in this manuscript goes as follows: first, experiments find that among the five lineages, there is one that will later dominate therefore becomes the MPC lineage; then a hypothesis is given based on a previous published paper (Geng et al. 2022), that cells may produce signals inhibiting other lineages; a threshold-based model is then proposed, that is, when any lineage population reaches this threshold, it will inhibit other lineages. The way that the model is designed (threshold-based) may trivialize the problem, as I believe the critical problem should be what makes this inhibition, and if you simply assume that the inhibition trivially exists in the largest lineage, it is not surprising that only this lineage dominates later. In that case, I do not see the value of the model here.

I think there are easy ways to dig deeper into the mechanisms while keeping the same model. For example, without any threshold effect but if all lineages are inhibiting each other, will this still

© 2025. Published by The Company of Biologists under the terms of the Creative Commons Attribution License (https://creativecommons.org/licenses/by/4.0/).

lead to the one-lineage-dominance later due to the stochasticity in cell divisions? I suggest the authors test this, and even it fails, it may help justify that some kind of threshold is necessary for the system. Overall, I think if the authors have to adopt the threshold setting, then they need to at least carefully discuss in their manuscript why such a threshold setting is a must-do.

2. Relate to the concern above, the main conclusion that is drawn from the current model is that the stochastic variation in cell division contributes to the dominance from one lineage. Then I suggest the authors to explore further into the effects of the stochasticity. Please try different values of \sigma, and examine how the noise strength will affect the dynamics. A thorough local or global sensitivity analysis would be even better, but at least some study in the noise strength \sigma should be provided.

I also have some questions on the biology side, but I apologize if it is because I didn't understand it well since I'm not a Ceratopteris expert:
1. Experimental results presented in Fig 9, 10 show that at the presence of Aphidicolin, the conversion to hermaphrodite can be delayed or even fully blocked. Such results are also tested in the math model. As I understand from the model, the only factor that is changed here is doubling the average cell dividing cycle, that seems like the same effect as slowing down the dynamics. Then, shouldn't this be equivalent to that in the baseline experiments, we cut the observation time earlier by half? If so, then it is not surprising that by the end of the half observation time, a lot of the conversions haven't been completed.

On the other hand, I'm curious about the ones that conversions are fully blocked. Taken into consideration of the dynamics being slowing down, what does it actually mean by "fully blocked" here? If we make the observation even longer, will they ultimately start conversion again? Or is there some other critical timepoint so that it has to at least start conversion before that, otherwise it will never convert any more? I hope the authors can clarify further on this.

2. The authors also discuss how periclinal and anticlinal divisions contribute to later lineage population distribution. In this system, is there a preferred type of divisions? Or is peri- vs. anticlinal divisions are mostly random? If possible, can authors give a count of peri- vs. anticlinal divisions?

Minor concern:

1. It is a little hard for me to understand the system when I read the Introduction, since I'm not familiar with Ceratopteris biology. Please consider include an illustration cartoon picture of the Ceratopteris, the male vs. the hermaphrodite, and the key organs that will be discussed later in the manuscript. A supplement Fig goes with the Introduction will greatly help people better understand the system.

First revision

Author response to reviewers' comments

Reviewer 1:

SUGGESTIONS TO AUTHORS

1) Many of the techniques and conclusions are similar to the results of Geng et al., 2022. For example, both cases use the term "Meristem progenitor cell (MPC)", and describe different "Phases" of meristem growth (Phase I, II, III for Geng et al., and Phase I and II for Yang et al). It would be helpful to include a comparison of these terms and findings throughout the text, to understand how these two processes- meristem formation in the hermaphrodite gametophyte and meristem formation during the male-hermaphrodite transition- are similar and different, and emphasize which findings are novel.

Response: Thank you for the helpful comment. We have included more detailed comparisons of

© 2025. Published by The Company of Biologists under the terms of the Creative Commons Attribution License (https://creativecommons.org/licenses/by/4.0/).

these two different processes in the revised main text.

It now reads: *"Quantitative analysis of time-lapse imaging data has enabled us to identify the cellular behaviors driving the male-to-hermaphrodite conversion, and to compare these with the processes associated with normal male and hermaphrodite development. Male gametophytes lack a meristem (8, 14), and in the presence of antheridiogen, most or all cells in a male gametophyte ultimately differentiate into sperm-producing antheridia (Fig. 1H)(8). In contrast, in the absence of antheridiogen, we identified a single non-antheridium cell (Figs. 2-3, S14) within the male body that functions as the MPC. The MPC lineage sustains active cell division and exclusively contributes to the formation of the entire new meristem (Fig. 3). At a later stage of the sex conversion, the MPC lineage exhibits an accelerated proliferation rate, while other non-antheridium cell lineages lose division activity and become mitotically inactive (Fig. 4). Previous live-imaging studies beginning at 5 days after spore inoculation (5 DAI) identified two or three marginal cells that exclusively contribute to meristem formation in hermaphrodites (28). Future analysis performed earlier than 5 DAI would help determine whether these two or three marginal cells originate from a single MPC, similar to the MPC identified during the male-to-hermaphrodite conversion. Additionally, the spatial and temporal dynamics of cell divisions observed during de novo meristems formation suggest two distinct phases: Phase I and Phase II (Fig. 4). These phases appear comparable to the three developmental phases—initiation, transition, and maturation—previously identified during normal meristem development in hermaphrodites (28). Specifically, Phase I aligns with the initiation phase of normal meristem development, while Phase II encompasses both the transition and maturation phases categorized in the earlier study (28)."*

2) The introduction focuses primarily on the sex determination of the gametophyte. Since the paper describes the development and structure of the meristem, it would be useful to include a short paragraph about meristem structure (e.g. apical meristem, marginal/lateral meristem, single-celled vs multicellular meristem) in different ferns, and what has already been shown. *E.g. Geng et al., 2022, Wu et al., 2022a, Wu et al., 2022b, Bartz and Gola, 2018, Takahashi et al., 2009. Some of these papers were referenced, but the description is very vague (line 65- 66).*

Response: Thank you for your comment. We have revised the introduction to include more detailed descriptions on different types of meristems in fern gametophytes.

Now it reads: *"Gametophytes of diverse fern species develop distinct types of meristems, including apical cell (AC)-based meristems and multicellular meristems. The AC-based meristem comprises a wedge-shaped AC and its progeny, typically located at the apical region of the prothallus, driving cell proliferation. Multicellular meristems, by contrast, exhibit different morphology and organization, consisting of an array of actively dividing, rectangular cells with small dimensions. Among them, the multicellular apical meristem is located at the prothallus apex, while the multicellular marginal meristem, also referred to as the lateral meristem, marginal meristem, or notch meristem in previous studies, originates from one lateral side of the prothallus."*

3) Throughout the text, the authors describe the process of meristem formation. However, it is unclear to me what defines the "meristem". During its formation, is there a landmark feature (e.g. notch shape? Frequency of division?) that marks the transition from a forming meristem to a formed meristem? In the figures, meristems are marked with magenta arrowheads, however it is difficult to understand when the authors are able to classify the structure as a meristem. Perhaps a clear definition of the marginal meristem in the introduction would help clarify this.

Response: Thank you for your comment. Based on both cell morphology and division activity, we can define the boundary of a meristem in Ceratopteris gametophytes. Specifically, multicellular meristems in Ceratopteris gametophytes are composed of rectangular cells with small dimensions, where each cell is largely occupied by a nucleus and exhibits continuous division activity. In contrast, cells outside the meristem are significantly expanded (as indicated in the figure), with their nuclei occupying only a small portion of the total cell volume. These cells also lose division activity, suggesting the onset of cell expansion and differentiation into parenchyma-like cells. We have included this information in the revised manuscript.

© 2025. Published by The Company of Biologists under the terms of the Creative Commons Attribution License (https://creativecommons.org/licenses/by/4.0/).

Now it reads: *"Specifically, the newly formed multicellular meristems in the converted hermaphrodites (Figs. 2V, S4V, S5X) were characterized by distinctive cell morphology and division activity. Multicellular meristems in Ceratopteris gametophytes are composed of rectangular cells with small dimensions, where each cell is largely occupied by a nucleus (Figs. 2V, S4V, S5X) and exhibits continuous division activity (Figs. 4, S8, S9). In contrast, cells outside the meristem are significantly larger, with nuclei occupying only a small portion of the total cell volume (Figs. 2V, S4V, S5X), and these cells lose division activity (Figs. 4, S8-S9)."*

4) Does the gametophyte prothallus only consist of one cell layer? This is implied by the type of analysis that is performed, but isn't explicitly stated. This could be added to the paragraph suggested in comment 2.

Response: Thank you for your comment. Yes, a Ceratopteris prothallus consists of a single layer of cells, as demonstrated in supplementary movies 1 and 2 from our previous publication (Geng et al., 2022 Communications Biology). This single-cell-layer structure is well-suited for the 2D image analysis platform we have been using. On the other hand, mature antheridia forms a complex 3D structure, and for this reason, we excluded them from cell division quantifications. We have revised the manuscript to clearly include and describe this information.

Now it reads: *"Because the meristem and prothallus of a hermaphrodite consist s of a single layer of cells, which is well-suited for non-invasive live imaging, a recent study using time-lapse confocal imaging revealed the cell division dynamics driving meristem development in Ceratopteris hermaphrodites (28)."*

"mature antheridia form a complex 3D structure containing multiple sperm nuclei (as shown in Fig. 2D-N), which were not included in the segmentation or division quantification in this study."

5) Line 119-121: Why were new reporter lines generated? What advantage do these lines have over previously described reporter lines? Since these lines are new, it would be good to include a description and photos of how these lines grow in comparison to wild type. Do they have any developmental defects?

Response: Thank you for these comments. First, the new *pCrUBQ10::H2B-GFP::3'CrUBQ10* reporter shows generally very bright signals, likely due to the activity of the *CrUBQ10* promoter. Furthermore, as reported in the previous study (Geng et al., 2022 Communications Biology), the *pCrHAM::H2B-GFP::3'CrHAM* reporter exhibits ubiquitous expression signals in prothalli (ideal for studying meristem cell dynamics) but is not expressed in the gametes of males or hermaphrodites, In contrast, the *pCrUBQ10::H2B-GFP::3'CrUBQ10* reporter shows bright signals in gametes of both antheridia and archegonia (Figs. 2-3). These features make the new reporter particularly suitable for this study, and we have included this discussion in the revised manuscript.

It now reads: *"Notably, both the previously published pCrHAM::H2B-GFP::3'CrHAM reporter (28) and the pCrUBQ10::H2B-GFP::3'CrUBQ10 reporter generated in this study exhibit ubiquitous expression signals in Ceratopteris prothalli. However, the pCrHAM::H2B- GFP::3'CrHAM reporter is not expressed in the gametes of Ceratopteris gametophytes (28), whereas the pCrUBQ10::H2B-GFP::3'CrUBQ10 reporter exhibits bright expression signals in the gametes from both antheridia and archegonia (Figs. 2, S2-S3), making it particularly well- suited for this study."*

Additionally, the *pCrUBQ10::H2B-GFP::3'CrUBQ10* transgenic lines do not exhibit any developmental defects. We have included a new figure with micrographs of gametophytes from the wild-type control and the *pCrUBQ10::H2B-GFP::3'CrUBQ10* transgenic line at the same DAG (Fig. S16).

6) Line 138-line 140: Live samples were imaged every 6 hours. Did imaging affect the rate of growth of the gametophytes?

Response: Thank you for your comment. We have included the following discussions in the

© 2025. Published by The Company of Biologists under the terms of the Creative Commons Attribution License (https://creativecommons.org/licenses/by/4.0/).

revised manuscript to address this comment.

"In the time-lapse experiments, gametophytes were imaged directly on their Petri dishes containing growth medium, without any mounting or dissection. After imaging, the samples, along with their Petri dishes, were promptly returned to the growth chamber adjacent to the confocal microscope and cultured under identical conditions until the next imaging time point. As demonstrated in our previous study (28), imaging at six-hour intervals effectively captured all division events in gametophytes, enabling comprehensive cell lineage analysis. Repeated observations from both prior (28) and current studies confirm that this imaging procedure does not interfere with normal division, growth, or differentiation of gametophytes. First, all live-imaged hermaphrodites developed fully expanded prothalli with distinct meristem notches and adjacent archegonia by the end of long-term live imaging experiments (28). Second, high cell division activity was consistently observed during the later stages of imaging (e.g., the yellow MPC lineages after 72 h in the three samples detailed in Supplementary Table S2), indicating that repeated imaging neither reduced cell division activity nor disrupted normal developmental processes."

7) Line 153-156: Are any more antheridia initiated after the transfer to antheridiogen free media? Or is it just that pre-existing antheridia are maturing?

Response: Thank you for your comment. Based on our observations, after being transferred to antheridiogen-free FM, the original male body primarily continued its pre-existing antheridium development program, completing the development of pre-determined antheridium cells rather than initiating many new antheridium mother cells. This observation is consistent with the multiple prior studies showing that antheridium initiation in fern gametophytes is predominantly induced by antheridiogen. We have included the revised description in the manuscript.

8) Line 268-274: For the computational modelling, four assumptions are made. Whilst the assumptions seem logical, they are not all experimentally validated. Is it true that anticlinal and periclinal divisions are equally likely? Could this be quantified from your existing cell division data? Could the authors show the results of the model if each of the assumptions was excluded?

Response: This is a great point. First, we have quantified the total numbers of anticlinal and periclinal division events in the outermost layer in the MPC lineage across three samples. As shown in newly added Table S4, there is no significant difference in the frequencies of anticlinal division and periclinal division. This information has been updated in the description of the model assumptions.

Now it reads: *"The anticlinal and periclinal cell division events during the male-to-hermaphrodite conversion were analyzed across three imaged samples (Supplementary Table S4). Given that the outermost layer is the primary driving force in de novo meristem formation, and the division orientation within this layer can be clearly categorized as either anticlinal or periclinal relative to the gametophyte boundary, every cell division event occurring in the outermost layer of the newly formed meristems was recorded for Samples 1-3 throughout the entire imaging period (Supplementary Table S4)."*

Second, in response to this comment and advice from Referee 3, we now compare the original version of our model to a new version of the model that implements inhibition in a different, but related, way. This allows us to unpack some of the choices involved in modeling inhibitory signals. Third, we have added a numerical study to determine the influence of two parameters (noise strength and ratio of division reduction for inner to outer cells) on our model findings. These new results appear in newly added Figures S11 and S12, and are discussed in the Discussion.

9) Line 362-366: The authors state that non-meristematic cells become meristematic during de novo induction of meristems. Is there a marker of differentiation that could be tracked after transfer to antheridiogen free media, to confirm if this is occurring in the gametophyte? Or

© 2025. Published by The Company of Biologists under the terms of the Creative Commons Attribution License (https://creativecommons.org/licenses/by/4.0/).

perhaps a stain for a component that may be present in differentiated cells but not meristematic cells?

Response: Thank you for your comment. We would like to provide more detailed description of this topic to address this comment. Based on previous characterizations in the literature (Hickok et al., 1987; Banks et al., 1993; Banks, 1999) and our own observations (Figure 1, Figure S1), male gametophytes do not have possess a meristem, whereas converted hermaphrodites from males develops a meristem. In the presence of antheridiogen, all or the majority of cells in a male gametophyte ultimately differentiate into sperm-producing antheridia without forming any meristem structures (Fig. 1H). In contrast, in the absence of antheridiogen, male gametophytes exhibit sustained active cell division (Fig. 1K), which eventually results in the formation of a new meristem (Fig. 1L). Lineage analysis demonstrated that all meristem cells originate from a single non-antheridium cell (designated as MPC) within the male gametophyte. It is a pity that there is no differential marker currently available to specifically label this cell. However, we have included confocal images with the DIC channel at 0 h to show the location and morphology the MPC (Fig. S14). These images demonstrate that the MPC does not belong to any potential apical cells or antheridium cells of the male gametophyte. In the revised manuscript, it reads:

"Male gametophytes lack a meristem (8, 14), and in the presence of antheridiogen, most or all cells in a male gametophyte ultimately differentiate into sperm-producing antheridia (Fig. 1H)(8). In contrast, in the absence of antheridiogen, we identified a single non-antheridium cell (Figs. 2-3, S14) within the male body that functions as the MPC. The MPC lineage sustains active cell division and exclusively contributes to the formation of the entire new meristem (Fig. 3). At a later stage of the sex conversion, the MPC lineage exhibits an accelerated proliferation rate, while other non-antheridium cell lineages lose division activity and become mitotically inactive (Fig. 4)."

10) Woudenberg et al., 2024 (Development, https://doi.org/10.1242/dev.203026) recently showed that auxin inhibits cell division in the fern gametophyte. Perhaps this paper should be cited in the discussion as a potential mobile inhibitory signal derived from the forming marginal meristem?

Response: Thank you for your comment. We have included this new finding into the discussion section.

Minor points:

11) Line 385: Give some examples of the signals from the epidermis in flowering plants.

Response: Thank you for your comment. We have included examples showing signals from the epidermis in the SAMs of flowering plants (Arabidopsis, for example). Specifically, based on the literature, microRNA394 moves from the epidermis to the inner cell layers to maintain stem cell functions. Additionally, microRNA171, which is synthesized in the epidermis, acts as a short-range mobile signal that regulates HAM location patterns along the apical-basal axis and the function of SAMs.

12) Methods: If possible, include the light spectra of the growth cabinets to improve experimental reproducibility. At the very least, record the total light intensity.

Response: Thank you for your comment. We quantified the light intensity (27±4 W/m^2) used for the time-lapse imaging and have included this information in the methods. The range of the light intensity we used is comparable to that reported in a previous study (Banks et al., 1993).

13) Line 466: "29C"-> "29°C"

Response: Thanks for this comment. We have revised this in the manuscript.

14) Figure 1: The frequency of conversion from male-hermaphrodite could be included here, to

© 2025. Published by The Company of Biologists under the terms of the Creative Commons Attribution License (https://creativecommons.org/licenses/by/4.0/).

show how reproducible the induction is.

Response: Thank you for your comment. We have included the numbers of samples analyzed and the number of samples displaying morphology comparable to the representative samples shown in Figure 1. Specifically, we observed that nine out nine males successfully converted into hermaphrodites after being transferred to antheridiogen-free fern medium.

15) Figure 2: Why are only two antheridia highlighted? Can't the same antheridia be marked as in Figure 3?

Response: Thank you for your comment.

While this work primarily focuses on de novo meristem formation rather than antheridium cell differentiation, we used white dashed circles in Fig. 2 to highlight two representative antheridia in the original male body, illustrating the developmental stages of their maturation until they ruptured and released sperm. Other antheridia followed a similar developmental process; however, labeling all antheridia in the time-lapse images would have made the images cluttered and difficult to follow.

In Fig. 3, we used single large dots outlined by dashed circles to represent the positions and lineages of all mature antheridia (that were not segmented) in the lineage maps, simplifying the visualization of complex 3D structures. As a result, it appears that more antheridia were marked in Fig.3 compared to Fig. 2. We apologize for any confusion this may have caused.

In response to your comment, we have revised Figs. 2-3 and Figs. S3-S7 to include white arrows indicating the same two representative antheridia in both the confocal images and the lineage maps. This adjustment ensures consistency and clarity across the figures.

16) Figure 3: The grey antheridium in Panel O disappears from panel P onwards. The rest are present throughout. Is there a reason for this?

Response: Thank you for your comment. Based on the confocal images (Fig. 2O-P), the antheridium you mentioned likely ruptured and began releasing sperm. It is common for GFP signals in mature antheridia to weaken or become invisible after rupture and cell death. Although we captured signals from several other antheridia post-rupture, the signal from the remaining nuclei of this antheridium was below the detection threshold in our confocal images, making it unavailable for subsequent segmentation and lineage analysis.

This is why the gray solid circles disappear in Fig. 3P and the subsequent panels of Figure 3. Since our study excludes mature antheridium cells from the quantitative division analysis and does not investigate antheridium development but rather focuses on cell lineages and dynamics during the de novo formation of new meristems from non-antheridium cells, we believe that the weakening or disappearance of signals during the normal antheridium maturation process does not impact the primary findings or conclusions of this work.

In this revise manuscript, it now reads: *"As part of normal developmental program, mature antheridia eventually rupture and release sperm (Fig. 2). After rupture, the GFP signals from the remaining nuclei in antheridia become weak or undetectable for image analysis. Additionally, GFP signals from released sperm were not included in image analysis or quantification (as shown in Figs. 2-3)."*

17) Figure 6: Label Sample 1, 2 and 3 as in other figures.

Response: Thank you for your comment. We already labeled these samples in the revised Figure 6, where we first present the periclinal division in the MPC first, followed by the anticlinal division in the MPC in the other two samples.

18) Figure 7: The figure legend is very repetitive. Is there a more concise way to organise it?

© 2025. Published by The Company of Biologists under the terms of the Creative Commons Attribution License (https://creativecommons.org/licenses/by/4.0/).

Response: Thanks for this comment. We have revised the figure legend to make it more concise.

19) Figure 9: D-F in the figure show conversion to hermaphrodites, but the legend says they are remaining as males (line 751). Swap either the order of the panels or the text.

Response: Thanks for this comment. We already revised the figure legend.

20) Figure 10: The dashed circles are very thin and difficult to see. Increase the line thickness.

Response: Thank you for your comment. We have revised Figure 10.

Reviewer 2:
SUGGESTIONS TO AUTHORS

Some suggestions and Clarifications:

1. Antheridiogen promotes the formation of antheridia (male organs), which lack meristem. In the absence of antheridiogen, antheridia gain the ability to regenerate the meristem, leading to expansion of prothallus and the production of both egg-bearing archegonia (female organs) and antheridia. This reviewer is not clear whether hermaphrodites produce antheridia before archegonia or simultaneously. The conversion of the male organ into a hermaphrodite appears to resemble the process of an arrested hermaphrodite resuming its development to complete its growth. Whether or not this is the case, it would be helpful for readers to better understand the discoveries if the authors could compare these two developmental processes.

Response: Thank you for your comment. We have expanded the discussion on normal hermaphrodite development and the male-to-hermaphrodite conversion, to better describe these two processes. We also have included a diagram (Fig. S1) to illustrate the development of normal males and hermaphrodites, as well as the conversion process from males to hermaphrodites.

Based on the literature and our own observations, hermaphrodites develop a meristem that sustains continuous cell division and triggers the development of multiple archegonia adjacent to the meristem notch (Banks, 1999; Geng et al., 2022). Both the meristem and archegonia are regarded as female traits. Furthermore, the meristem of the hermaphrodite suppresses the formation of sperm-producing antheridia within or near the meristem. Consequently, only a few antheridia are formed at a distance from the meristem, typically near the spore coat or in the wing regions (Banks, 1999). Hermaphrodites usually differentiate one or two antheridia when they are forming archegonia within an overlapping developmental window (Banks, 1999). In contrast, male gametophytes exposed to antheridiogen only retain male traits, producing many antheridia without forming female traits such as a meristem or archegonia. However, in the absence of antheridiogen, non-antheridium cells in male gametophytes can undergo a cell fate change, forming meristems and archegonia, thereby acquiring female traits.

2. Fig. 1 shows that hermaphrodites exhibit a heart-shaped prothallus at 15 DAG (Fig. 1D). When a male gametophyte (2 DAG) was transferred to an antheridiogen-free growth medium, it seems taking a longer time to form a heart-shaped prothallus, as suggested by the difference in prothallus size when comparing images in Figs. B and C to those in Figs. J and K. This should be clarified.

Response: Thank you for your comment. Yes, the prothallus of the converted hermaphrodite (Fig. 1L) is indeed smaller than that of a hermaphrodite specified directly after germination (Fig. 1D). This size difference between the hermaphrodites arising from these two distinct processes is normal and consistent across all the samples we have observed. After germination, a hermaphrodite develops a meristem at approximately 2 days after germination (DAG). From that point, it undergoes rapid proliferation driven by active cell division in the meristem, resulting in a fully expanded prothallus within 10–12 days. In contrast, under the influence of antheridiogen, a male does not develop a meristem or exhibit active cell division, leading to a

© 2025. Published by The Company of Biologists under the terms of the Creative Commons Attribution License (https://creativecommons.org/licenses/by/4.0/).

much smaller prothallus with many differentiated antheridia (Fig. 1E–H). In the absence of antheridiogen, a male can convert to a hermaphrodite, but this process takes additional time. Normally, hermaphrodites display a heart-shaped prothallus by 6 DAG (Fig. 1B) and continue growing thereafter. However, during the male-to-hermaphrodite conversion, samples remain male at 6 DAG (Fig. 1J). After this point, a male begins initiating a new site of active proliferation (as shown in Fig. 1K), leading to the de novo formation of a meristem and the development of a new hermaphrodite body by 15 DAG (Fig. 1L). This new hermaphrodite body is smaller than a continuously proliferating hermaphrodite that has been developing since 2 DAG (Fig. 1A, D). We have revised the manuscript to clearly describe these processes.

3. In Fig. 2., please define how the egg-producing archegonia were defined.

Response: Thank you for your comment. As described in a previous study (Geng et al., 2022), the morphology and division patterns of cells during the initiation and proliferation of archegonia differ from those of other cell types. To provide a clearer depiction of developing archegonia in the converted hermaphrodite, we have included zoomed-in confocal images of GFP signals together with the DIC channel, which shows cell outlines, in Fig. S15.

In the revised manuscript, it reads: *"The morphology and division patterns of cells during the initiation and proliferation of egg-producing archegonia were distinct from those of other cell types in converted hermaphrodites. Zoomed-in confocal images of GFP signals together with the DIC channel (showing cell outlines) are included in Fig. S15, depicting developing archegonia in a converted hermaphrodite."*

4. This reviewer may have missed key details in the manuscript, so it would be helpful if the authors elaborate on how the MPC and cell lineages were defined in Figs. 3 and 4.

Response: Thank you for your comment. We first performed two-dimensional (2D) image analyses on the time-lapse imaging data. Each nucleus, identified by GFP signals in the images, was segmented and assigned a unique ID, with its position marked by a solid circle. Using these segmented nuclei, we generated dynamic lineage maps at multiple time points. At the initial time point (0 h), all non-antheridium cells in the male were designated as progenitors of distinct lineages, with adjacent nuclei labeled in different colors to trace lineage development over time. Lineage tracking was based on observed cell divisions in the time- lapse confocal images (Fig. 2). Cells and nuclei within the same lineage were consistently labeled with the same color across all time points. Additionally, at 0 h, nuclei within each antheridium were grouped and labeled with uniform colors. At later time points, nuclei from the same mature antheridium were grouped into larger dots to simplify visualization of the 3D structure. Among all color-coded lineages (Figs. 3A-U), one lineage—highlighted in yellow— stood out by consistently expanding, increasing in cell number, and forming the largest sector (highlighted with yellow dashed circles in Figs. 3, S6-S7). This yellow lineage exclusively contributed to the cells that formed the newly developed meristem (Fig. 3U, S6U, S7W, indicated by magenta arrowheads), while no other lineages contributed to the new meristem (Figs. 3U, S7U, S7W). The progenitor of this yellow sector (Figs. 3U, S6U, S7W) was traced back to a single non-antheridium cell at the initial time point (indicated by open magenta arrows in Figs. 3A, S6A, S7A). We identify this cell as the Meristem Progenitor Cell (MPC) in Fig. 3, which plays a critical role during the male-to-hermaphrodite conversion. We revised the main text for improved clarity regarding this process.

In Fig. 4, cells that underwent division during 12-hour intervals (0-12 h, 12–24 h, etc.) were labeled in magenta, while cells that remained undivided were labeled in green within the segmented images. To better illustrate the division activity of the MPC lineage over time, we have updated Figs. 4 and S8-S9 to specifically highlight the MPC lineage using yellow dashed circles.

It reads: *"During this phase, cell divisions became progressively restricted to the newly formed meristems (within the MPC lineage, highlighted with yellow dashed circles), reflecting the active proliferation occurring within these structures and driving their further development (Figs. 4E-J, S8E-J, S9F-K)."*

5. A couple of questions that are not required to secure the publication of this manuscript but

© 2025. Published by The Company of Biologists under the terms of the Creative Commons Attribution License (https://creativecommons.org/licenses/by/4.0/).

might be of interest: (1) Is the remaining portion of the male organ, without antheridia, still capable of converting into a hermaphrodite? (2) If the meristem is impaired, can hermaphrodites convert into antheridia?

Response: Thank you for your comments.

Regarding the first question, our observations indicate that after the male-to-hermaphrodite conversion, the remaining portion of the male organ generally cannot convert into a hermaphrodite. This is because, once a new meristem is formed de novo, it suppresses the formation of additional meristems from other non-antheridium cells.

For the second question, our previous study (Geng et al., 2022) demonstrated that when the meristem in a hermaphrodite is ablated, the remaining hermaphrodite develops a new meristem along with adjacent archegonia. This highlights the critical role of the meristem during the gametophyte phase in Ceratopteris.

In the main text, it reads: *"Geng et al. (2022) demonstrated that ablating the developing meristem notch in Ceratopteris triggers new meristem formation in another region of the gametophyte. One explanation for this observation is that meristem cells could produce an inhibitory signal that prevents cells in other lineages from actively dividing and initiating new meristems (28). Such a signal could be generated, for example, in actively dividing marginal cells."*

Reviewer 3:

1. I have concerns on how the modeling results support the experimental findings. As is pointed out by the authors themselves, the model used here is a simple one - which is fine with me, as long as the model can help elucidate the underlying mechanisms of the system. I feel like the model here is designed with the only goal that it behaves the same as what is observed from experiments, but very little efforts are made to touch the mechanisms behind. To be specific, the logic of the research in this manuscript goes as follows: first, experiments find that among the five lineages, there is one that will later dominate therefore becomes the MPC lineage; then a hypothesis is given based on a previous published paper (Geng et al. 2022), that cells may produce signals inhibiting other lineages; a threshold-based model is then proposed, that is, when any lineage population reaches this threshold, it will inhibit other lineages. The way that the model is designed (threshold-based) may trivialize the problem, as I believe the critical problem should be what makes this inhibition, and if you simply assume that the inhibition trivially exists in the largest lineage, it is not surprising that only this lineage dominates later. In that case, I do not see the value of the model here.

Response: We appreciate these helpful comments, and we agree that threshold-based behavior is a strong modeling assumption. We chose this approach because prior work on hermaphrodite gametophytes (Geng et al. 2022) suggests that ablation of the meristem can trigger mitotically inactive cells to apparently shift to high division, creating a new meristem. On the other hand, ablating the mitotically inactive cells had no detectable effect on the meristem cells. One way to model this is through a threshold describing switching behavior, but this raises the question of what mechanisms give rise to this threshold.

Following the referee's advice below, we now consider two different versions of our model (one with a threshold, and one without). We see these models as providing complementary insight into how inhibitory signals can reinforce a leading lineage that initially arises from stochastic division. We are very grateful to the referee for their suggestions that have improved our manuscript.

I think there are easy ways to dig deeper into the mechanisms while keeping the same model. For example, without any threshold effect but if all lineages are inhibiting each other, will this still lead to the one-lineage-dominance later due to the stochasticity in cell divisions? I suggest the authors test this, and even it fails, it may help justify that some kind of threshold is necessary for the system. Overall, I think if the authors have to adopt the threshold setting,

© 2025. Published by The Company of Biologists under the terms of the Creative Commons Attribution License (https://creativecommons.org/licenses/by/4.0/).

then they need to at least carefully discuss in their manuscript why such a threshold setting is a must-do.

Response: We appreciate this very helpful advice. Yes: in our revised manuscript and supplementary material, we now include results from the original version of our model and a new version of the model that does not include a threshold. Our new “mutual inhibition” version of the model confirms that all-lineage inhibition without a threshold can lead to one-lineage-dominance. We have chosen to keep both models in the study because they offer slightly different perspectives on differentiation and division activity. These changes appear at lines 285–300 and at lines 665–688. In Figure S12 and the Discussion, we highlight that the two models also behave differently under reduced noise strength, and we suggest considering additional alternative methods for incorporating inhibition into the model as a useful direction for future work (changes at lines 457–486 in the Discussion).

2. Relate to the concern above, the main conclusion that is drawn from the current model is that the stochastic variation in cell division contributes to the dominance from one lineage. Then I suggest the authors to explore further into the effects of the stochasticity. Please try different values of \sigma, and examine how the noise strength will affect the dynamics. A thorough local or global sensitivity analysis would be even better, but at least some study in the noise strength \sigma should be provided.

Response: This is a good point, and we have added a new figure in the supplementary material to account for it. First, Figure S12A presents the difference between the average cell number trajectories *in silico* and *in vivo* as a function of different noise strengths. This graph shows that noise strength affects the dynamics, and that our chosen noise strength is near the minima for the range that we considered. Second, Figure S12B presents a graph showing the error between the average *in vivo* and *in silico* trajectories as a function of the reduction parameter γ that describes differences in division rate between outer and inner cells. Notably, we find that both versions of our model are robust to this parameter as long as it is sufficiently low. We highlight this now in the Discussion at lines 432-434. Third, Figure S12C-D presents heatmaps of the error between the average *in vivo* and *in silico* trajectories as a function of noise strength and the reduction parameter γ, for the two different versions of our model. In Figure S12E-F, we show example mean simulated results under different noise strengths. Notably, we find that doubling the noise strength leads to the same mean qualitative behavior of one lineage emerging as the leader. However, setting the noise strength near zero disrupts the development of an MPC lineage in the timeframe that we consider. We now highlight this result in the Discussion at lines 472–482. While a thorough sensitivity analysis is out of the scope for our current initial step toward using modeling and experiment to elucidate cellular and molecular signals in Ceratopteris, we mention this as a place for further work and improvement in the revised Discussion at lines 480–482.

I also have some questions on the biology side, but I apologize if it is because I didn't understand it well since I'm not a Ceratopteris expert:

1. Experimental results presented in Fig 9, 10 show that at the presence of Aphidicolin, the conversion to hermaphrodite can be delayed or even fully blocked. Such results are also tested in the math model. As I understand from the model, the only factor that is changed here is doubling the average cell dividing cycle, that seems like the same effect as slowing down the dynamics. Then, shouldn't this be equivalent to that in the baseline experiments, we cut the observation time earlier by half? If so, then it is not surprising that by the end of the half observation time, a lot of the conversions haven't been completed.

On the other hand, I'm curious about the ones that conversions are fully blocked. Taken into consideration of the dynamics being slowing down, what does it actually mean by "fully blocked" here? If we make the observation even longer, will they ultimately start conversion again? Or is there some other critical timepoint so that it has to at least start conversion before that, otherwise it will never convert any more? I hope the authors can clarify further on this.

Response: Thank you for this thoughtful comment. From an experimental perspective, we agree that extending the observation period may reveal that some of these six samples could begin the

© 2025. Published by The Company of Biologists under the terms of the Creative Commons Attribution License (https://creativecommons.org/licenses/by/4.0/).

conversion process, aligning with the other six samples that exhibited a reduced or slowed conversion process. To describe this finding more precisely and avoid any potential confusion, we have revised the term “full blocked” to “*the conversion of males to hermaphrodites was disrupted (Fig. 9D-F) or halted (Fig. 9G-I) during the time window (0-6 DAT) we examined.*” From the mathematical perspective, we have tested simulating our model for a longer period of time (not included in the manuscript), and yes, the mean cell trajectories indicate that an MPC lineage will eventually be established. These simulations also suggest that this MPC lineage will grow to unrealistically high cell numbers. We interpret this as meaning that our model is missing additional signals that regulate overall gametophyte growth at later times (both in the treated and untreated cases). To account for this in the manuscript, we now note in the Discussion at lines 482–483 that our model is only meant to account for cell behavior during the empirical measurement times, and we expect additional signals are active at later times.

2. The authors also discuss how periclinal and anticlinal divisions contribute to later lineage population distribution. In this system, is there a preferred type of divisions? Or is peri- vs. anticlinal divisions are mostly random? If possible, can authors give a count of peri- vs. anticlinal divisions?

Response: Reviewer 1 raised a similar question, and in response we calculated the total numbers of anticlinal and periclinal division events in the outermost layer in the MPC lineage across three samples. As shown in our newly added Table S4, there is no significant difference in the frequencies of anticlinal and periclinal division in the outermost layer among our samples. We now discuss this finding and refer to Supplementary Table S4 when we present our model.

Now it reads: “*The anticlinal and periclinal cell division events during the male-to-hermaphrodite conversion were analyzed across three imaged samples (Supplementary Table S4). Given that the outermost layer is the primary driving force in de novo meristem formation, and the division orientation within this layer can be clearly categorized as either anticlinal or periclinal relative to the gametophyte boundary, every cell division event occurring in the outermost layer of the newly formed meristems was recorded for Samples 1-3 throughout the entire imaging period (Supplementary Table S4).*”

Minor concern:

1. It is a little hard for me to understand the system when I read the Introduction, since I'm not familiar with Ceratopteris biology. Please consider include an illustration cartoon picture of the Ceratopteris, the male vs. the hermaphrodite, and the key organs that will be discussed later in the manuscript. A supplement Fig goes with the Intrdoduction will greatly help people better understand the system.

Response: Thanks for this comment. We have included a new diagram (Fig. S1) to provide a clearer depiction of the hermaphrodite and male gametophytes of Ceratopteris. This figure also labels all the different organs and illustrates the conversion from male to hermaphrodite in the absence of antheridiogen.

Second decision letter

MS ID#: dev.204411R1

MS TITLE: Single Cell Derived Multicellular Meristem: Insights into Male-to-Hermaphrodite Conversion and De Novo Meristem Formation in Ceratopteris

AUTHORS: Xi Yang, An Yan, Xing Liu, Alexandria Volkening and Yun Zhou

I am happy to tell you that your manuscript has been accepted for publication in Development, pending our standard publication integrity checks.

© 2025. Published by The Company of Biologists under the terms of the Creative Commons Attribution License (https://creativecommons.org/licenses/by/4.0/).

Comments from the Reviewers:

Reviewer 1: I thank the authors for their thoughtful and clear responses to my comments. The changes implemented address my prior concerns and improve the clarity of the manuscript and the strength of the conclusions. I really enjoyed the paper and commend the authors for their hard work in the revision!

Reviewer 3: I appreciate authors' great efforts in addressing my previous questions. I do not have further comments and I think the manuscript is in good shape to be published.

© 2025. Published by The Company of Biologists under the terms of the Creative Commons Attribution License (https://creativecommons.org/licenses/by/4.0/).
